# Deletion of Glutamine Synthetase Gene Disrupts the Survivability and Infectivity of *Leishmania donovani*

**DOI:** 10.3389/fcimb.2021.622266

**Published:** 2021-02-26

**Authors:** Vinay Kumar, Sanhita Ghosh, Kamalika Roy, Chiranjib Pal, Sushma Singh

**Affiliations:** ^1^ Department of Biotechnology, National Institute of Pharmaceutical Education and Research, Mohali, India; ^2^ Cellular Immunology and Experimental Therapeutics Laboratory, Department of Zoology, West Bengal State University, Barasat, India

**Keywords:** leishmaniasis, glutamine synthetase, knockout, essentiality, infectivity

## Abstract

Glutamine synthetase (GS) is one of the most important metabolic enzymes which catalyzes ligation of glutamate and ammonia to form glutamine. Previous studies from our lab had revealed significant differences in parasite and host GS enzyme which warranted us to further work on its relevance in parasite. To analyze glutamine synthetase function in *Leishmania*, we generated GS overexpressors and knockout mutants and evaluated their ability to grow *in vitro* in monocyte differentiated macrophage and *in vivo* by infections in BALB/c mice. GS knocked out strain showed significant growth retardation with delayed cell cycle progression and morphological alteration. Null mutants exhibited attenuated infectivity both in *in vitro* and *in vivo* experiments and the effect was reverted back when infected with GS complemented parasites. This indicated that the alterations in phenotype observed were indeed due to GS knockout. GS knockout also made the parasite increasingly sensitive to Miltefosine. Detailed investigation of mode of parasite death upon Miltefosine treatment by dual staining with Annexin-V conjugated FITC and propidium iodide, pointed towards apoptotic or necrotic mode of cell death. This is the first report to confirm that GS is essential for the survivability and infectivity of *Leishmania donovani*, and can be exploited as a potential drug-target.

## Introduction

Leishmaniasis is a major worldwide public health problem, with approximately 0.7 to 1.0 million new cases yearly and mortalities of around 20,000 to 30,000 annually (Burza et al., 2018; Valero and Uriarte, 2020). Visceral leishmaniasis (VL) is the most fatal form of leishmaniasis, caused by *Leishmania donovani* and *Leishmania infantum*. (Ready, 2014; Torres-Guerrero et al., 2017). VL is difficult to eradicate from the endemic areas as the vector control and chemotherapy have several limitations. The presently available treatment options are costly, have less efficacy, toxic to humans, have complex administration regimes and are faced with the challenge of resistance. (Uliana et al., 2018). Hence, it is necessary to look out for novel potential molecular or biochemical markers and design more specific and effective inhibitors based on the knowledge of molecular mechanisms employed by the parasite for its survival.

Glutamine synthetase (GS) is a key metabolic pathway enzyme that uses substrate glutamate and ATP to generate glutamine. Glutamine is a part of several metabolic processes which promote cell growth and proliferation. It also helps in the maintenance of acid-base homeostasis. Glutamine has been found to be important in cellular signaling pathways. It also supports infectivity of various pathogens (Newsholme et al., 2003; Haber et al., 2017; Cruzat et al., 2018). GS and its catalytic product glutamine are involved in regulation of several different enzymes such as tryptophan transaminase, asparagine synthetase, glucosamine synthetase, carbamoyl-phosphate synthetase, glutaminyl-tRNA synthetase, glutamate dehydrogenase and ornithine decarboxylase (Fisher, 1999; Burbaeva et al., 2005; Forde and Lea, 2007). In bacteria *Mycobacterium tuberculosis* and protozoan parasite *Plasmodium falciparum*, *in silico* methods identified GS as a viable drug target (Fatumo et al., 2009; Crowther et al., 2010). GS is essential for replication and survival of intracellular forms of *Trypanosoma cruzi* (Crispim et al., 2018). In *Leishmania chagasi*, GS stimulates the proliferation of T cells which results in elevation of interferon gamma (Martins et al., 2006). Inhibition of either TCA cycle or glutamine synthetase strongly inhibited amastigote growth and viability *in vitro* and in infected macrophages in *Leishmania mexicana* (Saunders et al., 2014). GS acts as a moonlighting protein in *Bacillus subtilis* and is involved in gene regulation in addition to its enzymatic function (Fisher, 1999). GS is functionally important in many pathogens and thus its viability as a drug target has been extensively explored. Implication of glutamine synthetase in pathogenesis and several metabolic processes makes it an attractive target for drug discovery.

*L. donovani* glutamine synthetase (*Ld*GS) exists as a single copy gene and the enzyme is expressed in promastigotes as well as in amastigote form (Kumar et al., 2017). Earlier studies from our lab had identified *Ld*GS as potential drug target for VL infections based on structural differences between the host and parasite enzyme indicating possibility of target based drug designing (Kumar et al., 2020). We had also reported the antileishmanial efficacy of methionine sulfoximine (MSO) and phosphinothricin (PPT, Glufosinate) which are well-known glutamine synthetase inhibitors (Kumar et al., 2017).

The current study focuses to explore the essentiality of the GS gene for parasite survival and infectivity. For this we performed targeted gene replacement of *Ld*GS to generate knockout strains and also generated GS overexpression strains. We report that *Ld*GS is vital to growth, *in vitro* and *in vivo* parasite infectivity and in cell cycle progression. Apart from this, deletion of GS from the parasite resulted in increased sensitivity to standard antileishmanial drug, miltefosine. The current study validates GS as an enzyme essential for parasite. Hence, targeting this important enzyme could be a promising approach for developing much-needed new inhibitor aimed at controlling *Leishmania* infection.

## Materials and Methods

### Materials

Gel extraction and plasmid isolation kits, RPMI 1640 medium without L-glutamine, ampicillin, phleomycin, streptomycin sulfate, Anti-Rabbit IgG antibody conjugated with alkaline phophatase were procured from Sigma-Aldrich, St. Louis, MO, USA. Rabbit was used for raising the customized antibody-polyclonal anti-*Ld*GS (Abgenex, Bhubaneswar, India). Hygromycin B and Geneticin (HiMedia), propidium iodide, Pure Link genomic DNA and RNA isolation Kit (Invitrogen™), Annexin V-FITC apoptosis detection kit (Roche) and Poly-L-lysine coated coverslips was from Corning^®^ BioCoat™. The other chemicals used in the study were available commercially and were of analytical grade.

### Plasmids and Strains

*L. donovani* wild type (MHOM/IN/80/Dd8) promastigotes were used in the present study. Vector psp72αhygroα was a kind gift from Prof. Rentala Madhubala, Jawaharlal Nehru University, New Delhi, India. *Leishmania* specific expression vectors (pXG B3318, pXG B1288 and pXG B3324), were kindly provided by Dr. Stephen M. Beverly, Washington University Medical School, USA. These vectors were used for making knockout and complementation constructs.

### Culture Conditions of Parasite and Mammalian Cells

The promastigotes were grown in RPMI-1640 (pH-7.2) media supplemented with 10% Fetal Bovine Serum. It contained antibiotics- penicillin G, Streptomycin, Gentamycin (100 µg/ml each) and 0.2% sodium bicarbonate. They were cultured at 24°C. The monocytes THP-1 were maintained at 37°C, 5% CO_2_ environment in the same media.

For selection of the knockout, overexpressor and vector control, parasites were grown in complete RPMI media supplemented with respective antibiotics, *i.e.* Geneticin G418 (80 μg/ml), Hygromycin B (100 μg/ml) and Phleomycin (25 μg/ml). THP-1 monocytes were also maintained in RPMI 1640 media supplemented with 10% FBS in the humidified atmosphere with 5% CO_2_ and constant temperature of 37°C.

### Generation of Molecular Constructs for GS Gene Overexpression, Knockout and Complementation Studies

*Ld*GS open reading frame (Accession No. KT907048) was cloned in *Leishmania* specific episomal vector psp72αhygroα between *Xba*I and *Hind*III restriction site using gene specific primers H and I as mentioned in (**Table S1**). Positive clones were confirmed by colony PCR and double digestion of clones. The construct generated was verified by automated DNA sequencing (1^st^ BASE, Axil Scientific Pvt. Ltd, Singapore).

Knockout constructs were generated by cloning the upstream (5′ UTR of ~737 bp) and downstream (3′ UTR of ~785 bp) flanking regions of *Ld*GS present on chromosome number 6. The regions upstream and downstream of the gene were selected and specific primers were designed for the amplification of selected regions. The sequences selected as UTR regions are provided in **Supplementary Figure S1**. The vectors used for the overexpression and knockout studies are provided in **Supplementary Figure S2**. The single knockout construct (SKO) was generated by cloning 5′ UTR and 3′ UTR regions in pXG Hygro vector. The restriction enzyme site of *Nhe*I and *Xho*I was selected for cloning of 5′ UTR-*Ld*GS upstream of hygromycin resistance cassette using primers A and C (as mentioned in **Supplementary Table S1**) to generate 5′ UTR-*Ld*GS-pXG Hygro construct. The confirmed construct (5′ UTR *Ld*GS-pXG Hygro) was further used to clone ~785 bp of 3′ UTR *Ld*GS using primers D and E (as mentioned in **Supplementary Table S1**) in between restriction sites of *Nsi*I and *Bam*HI respectively to generate SKO construct. The double knockout construct (DKO) was generated using pXG-Neo vector. The primers used to amplify the 5′ UTR *Ld*GS had *Sal*I and *Xho*I restriction sites. For 3′ UTR, the restriction sites were *Nsi*I and *Bam*HI for cloning in pXG-Neo vector to generate complete DKO construct.

To restore *Ld*GS in the knock out parasites complementation construct was generated, the *Ld*GS gene was amplified using sense and antisense primers F and G respectively (**Supplementary Table S1**) with *Bst*EII restriction enzyme site. The gene was cloned into pXG Phleo vector. To confirm the orientation and sequence, the constructs were subjected to automated sequencing.

### Generation of Genetically Manipulated Parasites

*L. donovani* GS knockout parasite was generated by transfection of all the constructs with a Bio-Rad Gene Pulsar apparatus and conditions used to electroporate the parasite were at 443 V, 500 µF capacitance, ∞ ohm resistance and 16 ms time constant (Cruz et al., 1991). The harvested cells were washed with PBSG buffer twice. This buffer consisted of 10 mM NaH_2_PO_4_, 10 mM Na_2_HPO_4_, 145 mM NaCl and 2% glucose. The cells were washed with electroporation buffer containing 21 mM HEPES, 137 mM NaCl, 5 mM KCl, 0.7 mM Na_2_HPO_4_, 6 mM glucose. The final resuspension of cells was done in 400μl electroporation buffer to which about ~12 µg of gene replacement SKO cassette linearized with *Nhe*I and *BamH*I (5′UTR *Ld*GS-pXG Hygro-3′UTR) was added along with the cells in the cuvette (2mm). It was kept in ice for 10 minutes and then electroporated. The cells were transferred to 5 ml of RPMI medium with 20% FBS and maintained at 24°C. Antibiotics were added 48 h post transfection for selection of transgenic parasites expressing respective antibiotic resistance gene (Dolai et al., 2008).

*Ld*GS heterozygous mutant (*Ld*GS^+/−^) were maintained in 100 μg/ml of hygromycin B. The *Ld*GS heterozygous mutant was transfected with *Ld*GS-pXG Phleo vector for episomal expression of GS gene to generate *Ld*GS complementation mutant (*Ld*GS^+/−/+^) which were maintained in 100 μg/ml of hygromycin along with 25 μg/ml of phleomycin. The *Ld*GS^(+/−)^ and *Ld*GS^(+/−/+)^ mutants were selected for the second round of transfection with *Bam*HI linearized DKO construct 5′ UTR-*Ld*GS-Neo-3′-UTR to replace second allele for null mutant (*Ld*GS^−/−^) and add back mutant generation (*Ld*GS^−/−/+^). *Ld*GS^(−/−)^ mutants were maintained in presence of 100 μg/ml of hygromycin and 50 μg/ml of G148 (Genetecin). Add back null mutants (*Ld*GS^−/−/+^) were maintained in 50 μg/ml of G148 (Genetecin), 100 μg/ml of hygromycin B and 25 μg/ml of phleomycin. The strategy for GS gene knockout is illustrated in **Supplementary Figure S3A**. GS null mutant failed to grow in media deprived with l-glutamine and could grow only when transferred to RPMI complete media supplemented with l-glutamine.

For overexpression of GS in *Leishmania* promastigotes (*Ld*GS^++/++^) transfection of the ~10 µg *Ld*GS psp72αhygroα construct was done through electroporation by same protocol. After selection, the mutant parasites were maintained under constant selection pressure of suitable antibiotic.

### Genotypic Confirmation of GS Knockout by PCR

Knockout strains were confirmed by isolating genomic DNA from wild type and all mutant transgenic parasites and PCR based strategy was employed to show that the gene replacement cassettes had integrated in the genome of the parasite. The primers used are mentioned in **Supplementary Table S2** and primer orientation is schematically represented in **Supplementary Figure S3B**. Correct integration of the *hygro^r^* or *neo^r^* gene cassette in heterozygous and null mutant strains was confirmed by PCR of genomic DNA using combination of Neo or Hygro specific sense and antisense primer and the primers for 5′ or 3′ untranslated regions were also used. The genomic DNA of the mutant parasites was subjected to PCR to confirm the presence of the following events: presence of *hygro^r^* gene, *neo^r^* gene, knockout of *Ld*GS, using primer combination of P3 + P4, P5 + P6, P1 + P8 and P7 + P2. While integration of 5′UTR to *hygro^r^* gene, 3′UTR integration to *hygro^r^* gene, 5′UTR integration to *neo^r^* gene, integration *neo^r^* gene and 3′UTR, *phleo^r^* in episomal construct was confirmed by using primer combination of P1 + P4, P3 + P2, P1 + P6, P5 + P2 and P9 + P10, respectively in separate reaction using genomic DNA as template from all mutant strains keeping wild type genomic DNA as control. Confirmation of complementation and replacement of *Ld*GS gene was also done by PCR.

### Reverse Transcriptase PCR of GS Knockouts

The total RNA from wild type and mutant parasites was isolated using Pure Link RNA isolation kit (Invitrogen) and Prime Script™ one step RT-PCR Kit was used to perform RT-PCR. Primers used to perform RT-PCR are represented in **Supplementary Table S3**. To remove DNA contamination, the RNA was treated with DNAse I enzyme (Thermo Scientific). The expression of *Ld*GS in wild type parasites was considered as the reference for normalization and *Ld*-actin was taken as control.

### Preparation of Crude Lysate and Determination of GS Enzyme Activity

The promastigotes were centrifuged at 3000g and the pellet was washed with PBS (pH -7.4) Cells were resuspended in lysis buffer (20 mM Tris (pH 7.8), 1 mM PMSF, 0.5 µg/ml each of aprotinin and leupeptin). The cells were lysed using repeated freeze-thawing (Padmanabhan et al., 2005). The lysate was centrifuged at 13,000g, 30 min at 4°C. The protein was estimated using Bicinchoninic acid method (BCA) taking BSA (bovine serum albumin) as standard (Smith et al., 1985). To determine the specific activity and to perform western blot, the above cell lysate was used. *Ld*GS enzyme activity was performed by inorganic phosphate (Pi) determination method (Gawronski and Benson, 2004).

Briefly, for measuring the enzyme activity of GS in crude lysate, the reaction mixture containing 5 mM MgCl_2_, 5 mM NH_4_Cl, 3 mM ATP and 20 mM L-glutamate in 20 mM Tris-HCl (pH 7.8) and 10 μg of lysate was kept for 20 min at temperature of 37°C. Reaction mixture without crude cell lysate was taken as blank. Blue colored phosphomolybdate complex formation was recorded at 655 nm according to the protocol described previously. Percentage change in specific enzyme activity of all mutant strains were calculated keeping wild type enzyme activity as control.

### Immunoblotting of *Ld*GS

The total cell protein was quantified by bicinchoninic acid method and 75 µg protein was run on 10% SDS PAGE and the resolved protein was transferred onto nitrocellulose membrane and blocked with 5% BSA and the membrane was probed with anti-*Ld*GS antibody (1:4000 dilution). The membrane was further incubated with anti-rabbit alkaline phosphatase conjugated secondary antibody (1:7500). The visualization was done using BCIP and NBT (Sigma) as substrate and densitometric analysis was carried out by using Image J software. Alpha tubulin was taken as endogenous control.

### Determination of Growth Rate and Infectivity

To analyse growth rate, 1 × 10^6^ cells/ml stationary phase cells were inoculated in 25 cm^2^ flasks containing RPMI complete medium without addition of particular antibiotic. Cells were maintained at 24°C and cell count of the flask were analyzed at fixed time interval of 24 h by cell counting using Neubauer hemocytometer under a compound microscope for a period of 5 days.

The THP-1 monocyte cells were differentiated into adherent macrophages using phorbol 12-myristate 13-acetate (PMA). Infection was performed taking the ratio of 1:10 (macrophage: parasites) of cells. Infection of stationary stage parasites (WT and mutant) was done for 24 hours to perform parasite rescue transformation assay (Jain et al., 2012). 2×10^5^ THP-1 cells were plated per well of a 96 well plate and differentiated using PMA at 37°C in CO_2_ incubator with 5% CO_2_ for 48 h. The adhered monocyte derived macrophage cells were infected with parasite as mentioned above. The non-internalized promastigotes were removed and infected adhered macrophages was washed with serum free media. After cell lysis, RPMI-1640 complete medium was added to each wells and kept at 24°C for 48 h so that the promastigotes would be transformed from amastigotes. Finally, MTT assay was performed as per the previous protocol and absorbance was recorded at 540 nm (Mosmann, 1983). The percentage infectivity was calculated relative to wild type.

### Cell Cycle and Scanning Electron Microscopy (SEM) Analysis

*L. donovani* promastigotes were synchronized using hydroxyurea as per the protocol described earlier with minor modification (Pal et al., 2017). Briefly, 4×10^6^ cells/ml late exponential phase promastigotes were incubated in serum-deprived RPMI-1640 medium containing 100 µg/ml hydroxyurea (HU) for 8 h. After 8 h of cell synchronization, hydroxyurea was removed by low speed centrifugation at 1300g and washed with serum-free media and cultured in RPMI-1640 media (10% FBS) for 12 h and then washed with ice cold PBS. The pellet was resuspended by adding PBS, 300 μl followed by addition of 700 μl of chilled ethanol. and incubated at 20°C overnight. Ethanol was removed by centrifugation at 1300g and cells were washed with ice cold PBS. Next, samples were incubated for 15 min on ice with 500 µl solution containing 0.05% Triton X-100 and 100 μg/ml RNase A prepared in PBS. Propidium iodide (50 μg/ml) was used to stain the cells for 30 minutes and subjected to flow cytometry on FACS Aria Fusion (BD Biosciences)… At last, collection of 20,000 events, separately for each sample was done. The distribution of cells thus obtained were scrutinized using Flow Jo software. This was used to determine the percentage cells in each phase of cell cycle- G_0_/G_1_, S and G_2_/M.

To observe the effect of GS overexpression and deletion on the parasite morphology, SEM was used. 1x 10^6^ promastigote parasites were harvested and washed with ice-cold PBS. Cells were fixed in 2.5% glutaraldehyde (EM-grade glutaraldehyde, Sigma-Aldrich) containing 4% (w/v) paraformaldehyde in 0.1 M phosphate buffer. Cell suspension was added on L-lysine-coated coverslips (Corning^®^ BioCoat™) and incubated for 6 h at room temperature. Unadhered cells were removed and coverslip was washed with sterile filtered water to remove any buffer salts. To dehydrate, graded series of ethanol (v/v) 30%, 50%, 70%, and 90% were added in ascending order. The drying of samples was done at critical point, followed by their mounting on metallic stubs using bioadhesive carbon tape. Sputter coating of gold and palladium was done. The scanning electron microscope imaging was done using S-3400N,(Hitachi, Japan) (Gluenz et al., 2015; Soumya et al., 2017).

### Drug Sensitivity Assays

MTT (3-(4,5-dimethylthiazol-2-yl)-2,5-diphenyl tetrazolium bromide) assay was performed to assess the drug susceptibility for Miltefosine (standard anti-leishmanial drug) in wild type parasite and GS mutants (Mosmann, 1983). In a 96-well plate, 2×10^5^ cells were plated in each well followed by 48 h incubation at 24°C. Then, 10-100 µM of Miltefosine was added and incubated for 48h followed by addition of 20 µl MTT reagent of concentration 5mg/ml. The plate was incubated for 4 h at 37°C and then centrifuged at 3000g, 10 min. After the removal of supernatant, 100 µl of 100% DMSO was used to dissolve the formazan crystal. The absorbance was measured at 540 nm. The untreated cells were taken as control and IC_50_ value was calculated relative to it.

### Annexin V-FITC/PI Dual Staining for Determination of Apoptosis or Necrosis

In the event of early apoptosis, the phospholipids on the cell membrane are altered which leads to exposed phosphatidylserine on the surface of cell. In normal parasites, phosphatidylserine are not exposed, while in cells undergoing apoptosis phosphatidylserine are exposed to the cell surface (Mukherjee et al., 2018). 1 x 10^6^ cells/ml were plated in each well of a 24 well plate and kept at 24°C for 24 h. The late apoptotic cells bind to both AnnexinV/FITC and PI. The double positive population contains necrotic cells and can also have late apoptotic cells. Also, PI single positive events may represent pure nucleic acid aggregates. The promastigotes were treated with miltefosine and to detect apoptosis, AnnexinV-FLUOS kit (Roche) was used following the manufactures protocol. They were analysed using flow cytometry. All the knockout strains along with wild type cells (1 x 10^6^/ml) were seeded in 24 well plate and incubated at 24°C. After 24 h, untreated cell lines along with cells treated with IC_50_ concentration of miltefosine and N-acetyl cysteine preincubated cells treated with IC_50_ concentration of miltefosine were washed with cold PBS (pH 7.4) at 1300g. The resulting pellets were resuspended in 500 μl of staining buffer and incubated with annexin V-FITC first for 10 min followed by addition of PI. Cultures incubated at 45°C for 2 h were used as positive death controls. Prior to being subjected to flow cytometry, the samples were kept in ice (in dark) for 15 minutes and analyzed by flow cytometry (FACS Aria™ Fusion, Becton Dickinson) with BD FACS Diva™ software. Argon laser (excitation wavelength 488 nm) and 561 nm yellow-green laser was used for detection of Annexin V-FITC stained (early apoptotic) cells in (FITC; 530/30 band pass) filter and PI stained (late apoptotic or necrotic) cells in (PE/PI; 582/15 band pass) filter. 30 000 events in total had been taken for analysis. FITC log (x axis, FITC-fluorescence) versus PI/PE log (y axis, PI) were used for data acquisition. Data was analysed by FlowJo software, Ver. 10.3.1.

### Determination of Parasite Infectivity by the Mutant Parasites, *In Vivo*

For this experiment, male BALB/c mice were procured from Imgenex India, Bhubaneswar. They were kept at 25°C ± 2°C and subjected to a 12 h light dark cycle. They were fed *ad libitum* (Mukherjee et al., 2020). The experimental groups (5 animals in each group; 4-6 weeks age; weighing approximately 20g) were infected, i.v., with 2 × 10^7^ promastigotes of *Ld*GS^(+/+)^, *Ld*GS^(+/−)^, *Ld*GS^(+/−/+)^, *Ld*GS^(−/−)^, *Ld*GS^(−/−/+)^, *Ld*GS^(++/++)^, and *Ld*: psp (Vc). Four-week post-infected animals were sacrificed, amastigotes were calculated from the spleen and liver Giemsa-micrographs under phase contrast microscope (Zeiss Axioscope), and parasite burden was extrapolated using Stauber’s formula as described earlier (Dey et al., 2020). The survival percentage of promastigotes maintained under specific antibiotic surveillance was monitored in the flow cytometer (BD FACS Verse) using 7-Aminoactinomycin D (7-AAD) before *in vivo* infection (Sultana et al., 2018).

As we have focused on the capacity of proliferation and survivability of *Ld*GS^(−/−)^ mutants’ inside the host cells, the time dependent infectivity of *Ld*GS^(−/−)^ parasites was investigated and compared with the *Ld*GS^(+/+)^, *in vivo*, at day 15 and day 30 post infection. The mRNA was extracted from the spleens of the infected animals and converted to cDNA. The expression of kinetoplast DNA (kDNA) was evaluated by semi-quantitative PCR (*L. donovani* kDNA; Forward: 5′-AAATCGGCTCCGAGGCGGGAAAC-3′ and Reverse: 5′- GGTACACTCTATCAGTAGCAC-3′), normalized against equal expression of house-keeping gene GAPDH (murine GAPDH; Forward: 5´-GAGCCAAACGGGTCATCATC-3´ and Reverse: 5´-CCTGCTTCACCACCTTCT TG-3´) (Dey et al., 2020). The densitometric quantification was analyzed by Image Lab software and represented as mean ± SE from 3 animals per group. The differences in means among various groups were considered significant as per the one-way analysis of variance using GraphPad Prism v 6.0 software, *p < 0.038 and **p < 0.032.

## Ethics Statement

The experiments involving animals had been approved by the institutional Animal Ethics Committee, WBSU, Barasat [File no. WBSU/IAEC/2019-20/8(CP), dated 20.05.2019]. IAEC registration no. 1394/GO/Re/S/10/CPCSEA, dated 09/12/2016). The institutional guidelines for animals had been followed in performing this study.

## Statistical Analysis

The data obtained from indicated number of experiments has been interpretated as mean ± S.D. values. Student’s unpaired t-test was performed in Graph-Pad Prism software (GraphPad, San Diego, CA, USA) for statistical data analysis. The data that showed differences from groups were verified by ANOVA and p values of ≤ 0.05 were considered statistically significant. The levels of statistical significance were indicated as follows: *p ≤ 0.05, **p<0.01, ***p<0.001, **** p ≤ 0.0001 whereas for *in vivo* animal experimental data analysis only *p* value of ≤ 0.05 were considered significant.

## Results

### Generation of *Ld*GS-psp72αhygroα Construct for Overexpression of *Ld*GS in *L. donovan* is (*Ld*GS^++/++^) and Functional Characterization of GS Overexpressors

**Figure 1:**
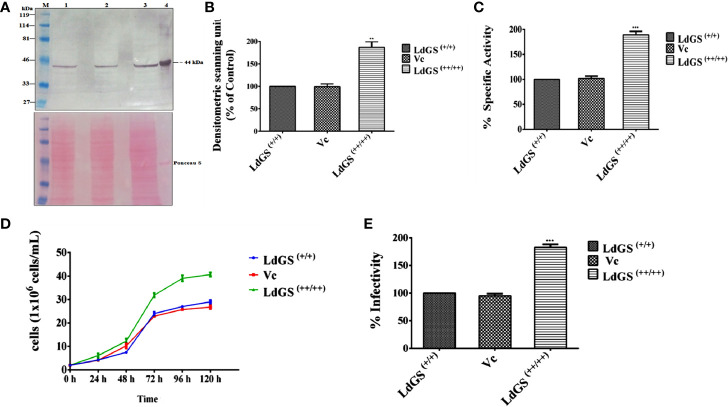
Confirmation of *Ld*GS overexpressor (*Ld*GS^++/++^) **(A)** Confirmation of *Ld*GS overexpression by western blotting. Top panel; Lane M: prestained broad range marker (Bio-Rad); Lane 1: crude cell extract of Wild type (WT) promastigote; Lane 2: crude cell extract of vector control (Vc); Lane 3: crude cell extract of GS overexpressor (*Ld*GS^++/++^); Lane 4: purified recombinant *Ld*GS protein. Bottom panel; The membrane is stained using Ponceau-S as a loading control; **(B)** The bar graph represents the densitometric analysis of the western blot represented in A; **(C)** Percentage GS activity was measured in total cell lysate Vc (*Ld*:psp) and *Ld*GS^(++/++)^ compared to wild-type *Ld*GS^(+/+)^, **(D)** Effect of *Ld*GS overexpression on parasite growth rate. Analysis of the growth rates of wild type, psp and overexpression promastigotes. **(E)** Influence of GS gene overexpression on the infectivity of *L. donovani* parasites *in vitro*. Data represents mean ± SD of three independent experiments. p values of ≤ 0.05 were considered statistically significant and levels of statistical significance were indicated as follows: **p ≤ 0.01 and ***p ≤ 0.001.

GS overexpressors were generated as described in methodology section. Western blot analysis of whole cell lysate indicated ~1.9 fold increased GS expression in *Ld*GS overexpressor [*Ld*GS^(++/++)^] compared to wild type [*Ld*GS^(+/+)^] and *Ld*: psp vector control (Vc). A poncaeu stained blot was kept as the loading control (**Figures 1A, B** **)**. The ~1.87 fold higher enzyme specific activity by *Ld*GS^(++/++)^ over *Ld*GS^(+/+)^ and Vc confirmed the GS overexpression in the parasites (**Figure 1C**).

The growth of wild type [*Ld*GS^(+/+)^], *Ld*: psp vector control (Vc) and *Ld*GS overexpressor [*Ld*GS^(++/++)^] promastigotes were followed over five days. Counting of viable cells was done to monitor the growth rate of all transgenic cell lines. (**Figure 1D**). At 48 h, the parasite cell number was found to be 4 x 10^6^ in both the *Ld*GS^(+/+)^ and Vc however, *Ld*GS^(++/++)^ showed 6 x 10^6^ parasites. At 72 h, the cell number was found to be 24 x 10^6^, 22 x 10^6^ and 31 x 10^6^ respectively for the parasites. At 96 h, the *Ld*GS^(++/++)^ reached a cell density of 41 x10^6^ cells/ml whereas, *Ld*GS^(+/+)^ and Vc reached at a cell density of 29 x10^6^ cells/ml and 27.5 x10^6^ cells/mL respectively. No difference in growth was observed in *Ld*GS^(+/+)^ and Vc parasites up to 120 h. It was clearly observed that though there was no change in growth of *Ld*GS^(+/+)^ and Vc parasites yet the GS overexpressing parasites exhibited higher growth rate compared to the control parasite. The replication rate was observed higher in case of *Ld*GS^(++/++)^ promastigotes, indicating the involvement of GS in growth of *Leishmania*, at least, *in vitro*.

Next, to check the consequences of *Ld*GS gene overexpression on robustness of the over expressors in infecting host macrophages *in vitro*, the parasite rescue and transformation assay was performed with wild type [*Ld*GS^(++)^], vector control (Vc) and *Ld*GS overexpressor [*Ld*GS^(++/++)^] promastigote parasites. Phorbol-12-myristate-13-acetate (PMA) differentiated THP-1 cells were infected with wild type, vector control and GS overexpressor promastigotes for 24 h. Non internalized cells were removed and infected macrophages were washed with serum free media. Infected monocyte differentiated macrophage cells were lysed and incubated in complete media for 48 h at 24°C for performing the transformation assay. It was observed that there was no change in the intracellular survival of amastigotes in case of *Ld*GS^(++)^ and Vc. However, *Ld*GS^(++/++)^ parasites exhibited almost ~ 1.8 fold enhanced replication of intracellular amastigotes indicating the role of *Ld*GS in facilitating parasite replication within macrophages (**Figure 1E**). Thus, *Ld*GS overexpressor parasites exhibited higher infectivity rate to macrophages compared to wild type and vector control parasite. The result clearly indicated that GS affects infective ability of parasites.

### Nutritional Assessment of the *L. donovani* GS Mutant

The amino acid glutamine is an important component of several biochemical pathways such as protein synthesis. After glucose, it is also a source of energy for the cell. Comparing the growth rate of all knockout strains it was found that GS null mutant failed to grow in absence of glutamine, while growth rate of wild type and complementation mutant parasites was almost similar in glutamine or glutamine deficient media (**Supplementary Figure S4**). Add back null mutant growth rate was also very slow in glutamine deficient media. Indeed, when the mutant cells were transferred to glutamine supplemented media, even though impairment in *in vitro* growth was observed in case of null mutants but we were able to get sufficient cells to functionally characterize the role of GS gene in parasite.

### Genotypic Characterization of *Ld*GS Knockout Strains

**Figure 2:**
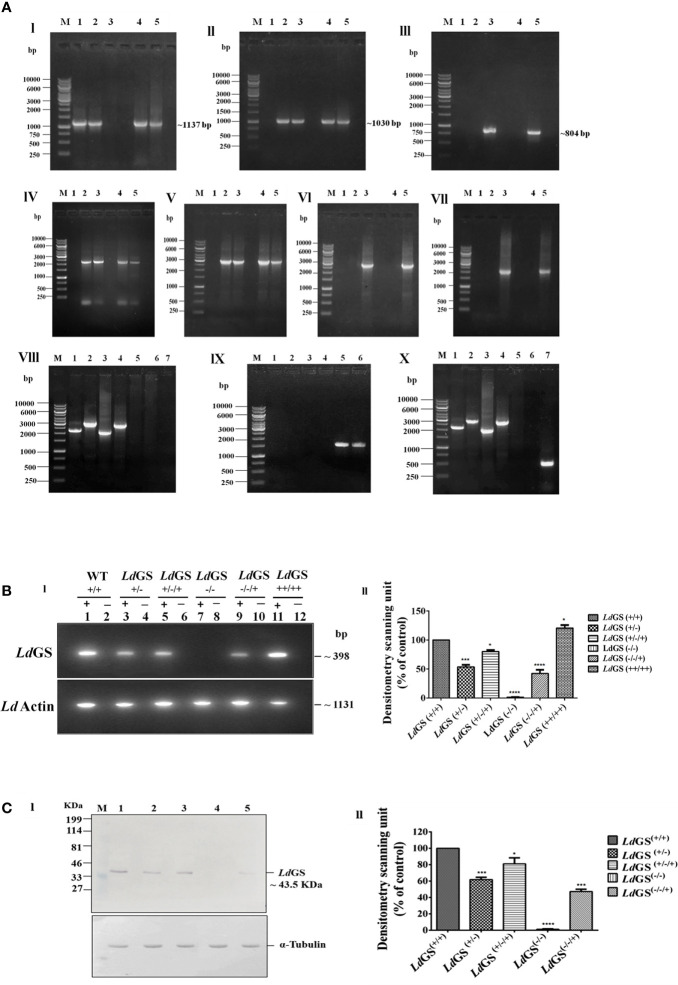
Confirmation of GS knockout. **(A)** Genotypic analysis of genetically manipulated promastigotes by PCR. Template for PCR analysis were the genomic DNA of the following: 1. wild type, *Ld*GS^(+/+)^; 2. heterozygous mutant, *Ld*GS^(+/−);^ 3. null mutant, *Ld*GS^(−/−)^; 4. complementation mutant*, Ld*GS^(+/−/+)^; 5. add back null mutant, *Ld*GS^(−/−/+)^ (I) Primers encompassing complete *Ld*GS ORF was used in the amplification of GS gene (~1137 bp) (II) The amplified product of primer P3+P4 to show presence of hygromycin resistance gene (~1030 bp) (III) primer P5+P6 to show presence of neomycin resistance gene (~804 bp) (IV)) primer P1+P4 to show integration of 5′ flanking sequence to hygromycin gene(~2189 bp). (V) primer P2+P3 to show integration of hygromycin to 3′ flanking sequence (~2961 bp). (VI) primer P1+P6 to show integration of 5′ flanking sequence to neomycin gene (~2001 bp) (VII) primer P2+P5 to show integration of neomycin to 3′ flanking sequence (~2775 bp). (VIII) Genomic DNA from GS null mutant cells were taken as PCR template. Internal primers specific to hygro, neo and wild type were used to validate the recombination. The specificity of recombination event was checked with Hygro, Neo and *Ld*GS (WT) gene specific internal primers. M: indicates the DNA molecular size marker in kb. Lane 1: amplified product of P1+P4; Lane 2: amplified product of P3+P2; Lane 3: amplified product of P1+P6; Lane 4: amplified product of P5+P2; Lane 5: amplified product of P1+P8 (5′UTR-*Ld*GS sense and *Ld*GS internal antisense primer); Lane 6: amplified product of P7+P2 *Ld*GS internal sense and 3′UTR-*Ld*GS antisense primer; Lane 7: negative control without DNA template (IX) Genomic DNA from wild type promastigotes was used as a template for PCR analysis with Hygro, Neo and *Ld*GS gene specific primer. M: denotes 1 Kb DNA ladder. molecular size marker in kb. Lane 1: amplified product of P1+P4; Lane 2: amplified product of P2+P3; Lane 3: amplified product of P1+P6 primer; Lane 4: amplified product of P2+P5; Lane 5: amplified product of P1+P8; Lane 6: amplified product of P2+P7 primer (X) PCR analysis of add back null mutant parasites using genomic DNA. To validate the recombination event, primers specific to hygro, neo, wild type and phleo were used., M: represents the DNA ladder. Lane 1-6: same set of primers in respective lane as represented in IX; Lane 7: amplified product of phleo gene to show presence of episomal construct using primer set P9+P10. **(B)**. RT-PCR of total cell RNA from genetically manipulated parasites. (I) Expression analysis of *Ld*GS mRNA in wild type (*Ld*GS^+/+^), GS heterozygous (*Ld*GS^+/−^), complementation mutant (*Ld*GS^+/−/+^), null mutant (*Ld*GS^−/−^), add-back null mutant (*Ld*GS^−/−/+^) and *Ld*GS overexpressor (*Ld*GS^++/++^) in *L. donovani* promastigotes using semi-quantitative reverse transcription PCR. Internal primer specific for the gene were used.(lanes have been marked as ‘+’). The negative control reactions are marked as ‘−’. The product sizes of (~398 bp) is indicated. As an endogenous control of *L. donovani* Actin gene was also amplified (1131bp). The bar graph shows the relative expression of GS using densitometric analysis of each sample. The house keeping actin gene was the control. The expression of GS in wild type was consider as standard for data normalization. The significance differences in expression are marked using asterisks. Two independent experiments were performed and the erroe bars show the mean ± SD value. **(C)** Analysis of *Ld*GS protein expression using immunoblots. (I) Lane M: prestained broad range marker (Bio-Rad); Equal amount of total cell proteins (100 μg) from Wild type (Lane 1), *Ld*GS^(+/−)^ (Lane 2), *Ld*GS^(+/−/+)^ (Lane 3); *Ld*GS^(−/−)^ (Lane 4); *Ld*GS^(−/−/+)^ (Lane 5) detected using anti-*Ld*GS antibody. Alpha tubulin was used as loading control. (II) A bar graph representing the densitometry analysis of the western blot is represented in **(C)** Data represents mean ± SD of two independent experiments. p values of > 0.05 were considered statistically non significant and levels of statistical significance were as follows: *p ≤ 0.05, ***p ≤ 0.001 and ****p ≤ 0.0001.

The genotype of GS knockout strains was confirmed by performing PCR using different sets of primers (**Supplementary Table S2**). The template used in the PCR analysis was the genomic DNA of WT parasites with combination of Hygro, Neo and *Ld*GS gene specific primers to show the absence of *hygro^r^* and *neo^r^ gene* in wild type genomic DNA. Primers 1 and 4 amplified ~2189 bp region of 5′UTR GS and *hygro^r^* gene while primers 2 and 3 covered the region of 3′UTR and *hygro^r^* gene with an amplified product of length ~2961 bp from genomic DNA of all mutant promastigotes. No amplified product was obtained from wild type genomic DNA using same set of primer combination which confirmed the integration of the hygromycin cassette in *Ld*GS^(+/−)^. Primers 1 and 6 encompassing 5′UTR and neomycin resistance gene internal region gave an amplified product of ~2001 bp size. Similarly, integration of *neo^r^* gene to 3′UTR *Ld*GS was confirmed using primers 2 and 5 which gives an amplified product of ~2775 bp size with *Ld*GS^(−/−)^ and *Ld*GS^(−/−/+)^ genomic DNA, while no amplified product was observed with wild type and *Ld*GS^(+/−/+)^ genomic DNA. This confirmed the replacement of second allele of GS gene by neomycin resistance cassette in *Ld*GS^(+/−)^. PCR analysis of the double antibiotic resistant strain with primers 1 and 8 along with 2 and 7 gave no amplified product with GS null mutant, while wild type genomic DNA showed amplified product of ~1,559 bp and ~ 1,500 bp respectively. This revealed that both hygro and neo cassettes replaced both the alleles of GS gene and also got integrated at the appropriate locus **[** **Figure 2A** (I–IX)]. P3 (Hygro sense) and P4 (Hygro antisense) pair of primer combination was used to show the presence of *hygro* ^r^ gene in *Ld*GS (all GS mutants except wild type) while other set P5 +P6 (Neo sense + neo antisense) combination was used to show the presence of Neo gene in null and add back null mutant cell line. A band of ~538 bp was obtained using primers P9 and P10 which are *phleo* specific internal primers thus confirming the presence of episomal *Ld*GS*-*pXG Phleo in the add-back null mutant cell line **(** **Figure 2A** X).

### Alteration in Expression of *Ld*GS mRNA in GS Knockout Strains by Reverse Transcriptase PCR

To confirm the expression of GS at the mRNA level, gene-specific sense internal primers were designed. Amplification was seen at ~398 bp in RT-PCR using gene specific internal primer as mentioned in methodology section. Negative control for each reaction did not contain any RNA template, which was included to show that the reaction system was not contaminated (**Supplementary Table S3** and **Figure 2B** top panel). Transcription of *Ld*GS gene was quantified by densitometric analysis of cDNA amplified product from all transgenic strains by Image J software (**Figure 2B** bottom panel). The results revealed significant reduction in GS mRNA levels of *Ld*GS^(+/−)^ and *Ld*GS^(−/−/+)^ strains. *Ld*GS^(++/++)^ showed ~ 20% higher level of mRNA expression while *Ld*GS^(+/−)^ mutant showed ~45.3% reduction and *Ld*GS^(−/−/+)^ mutant showed significantly higher level of 57.7% reduction in level of mRNA expression compared to *Ld*GS^(+/+)^. Null mutant (*Ld*GS^(−/−)^) showed no amplification. Reaction without RNA template also showed no amplified product.

### Confirmation of GS Gene Knockout by Western Blot Analysis

To check the alteration in *Ld*GS expression in all cell types: *Ld*GS heterozygous mutant, *Ld*GS complementation mutant, *Ld*GS null mutant and *Ld*GS add back null mutant western blot analysis was performed keeping expression of GS in wild-type as control. The NC membrane was incubated with the customized anti-GS antibody. Expression of GS in all transgenic promastigotes was observed by Western blot: wild type expressed endogenous levels of enzyme while *Ld*GS^(+/−)^ mutant showed reduction in GS expression by 38%, The protein expression in *Ld*GS complementation was similar to WT cells which implies the restoration of gene expression. Alpha tubulin was taken as endogenous control (**Figure 2C** I). Western blot analysis confirmed the almost complete loss of GS protein expression (~98%) in *Ld*GS null mutant promastigotes. Surprisingly, *Ld*GS^(−/−/+)^ add back mutant showed 67% reduction in protein expression compared to the wild type parasites **(** **Figure 2C** II).

### Functional Characterization of GS Mutant Cell Lines

**Figure 3:**
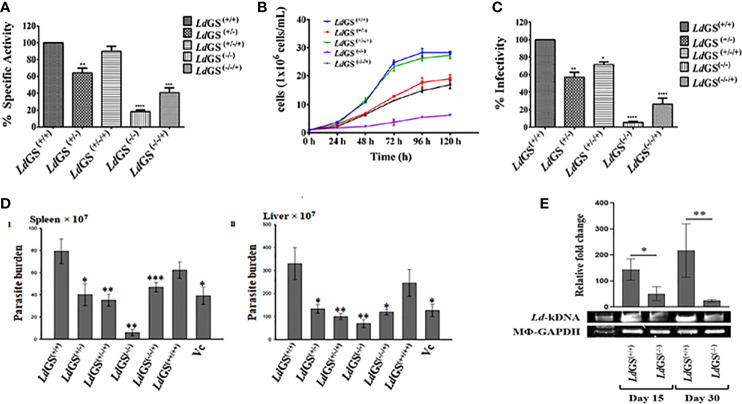
Characterization of knockout strains. **(A)** Confirmation of *Ld*GS gene knockout strains by enzyme activity. GS enzyme activity measured in total cell lysate extracted from wild type and different GS mutant cell lines by inorganic phosphate (P_i_) determination method, **(B)** Comparative Growth profile of wild type with GS heterozygous promastigotes, *Ld*GS^(+/−)^, complementation, *Ld*GS^(+/−/+)^, Null *Ld*GS^(−/−)^ and *Ld*GS^(−/−/+)^ add back null mutant promastigotes, **(C)** Effect of GS gene knockout on parasite infectivity. the percentage infectivity was analyzed for *Ld*GS mutant cells compared to wild type promastigotes. The Data has been calculated from three independent experiments and represented as mean± SD, **(D)** Effect of GS gene knockout on the infectivity of *L. donovani* amastigotes, *in vivo*: Burden of parasite in spleen and liver of the infected BALB/c mice with different mutant strains of *L. donovani* Dd8, *in vivo*. Data are representative of Mean ± SE from three independent experiments with 5 animals per group (*p < 0.002, **p < 0.001, ***p < 0.009, vs WT-infected control. **(E)** *Leishmania-*kDNA expression against the normalized equal expression of Mϕ-GAPDH in spleens of *Ld*GS^(+/+)^and *Ld*GS^(−/−)^ infected animals at day 15 and day 30, post infected. The densitometric quantification was analyzed by Image Lab software and represented as mean ± SE from 3 animals per group. The differences in means among various groups were considered significant as per the one-way analysis of variance using GraphPad Prism v 6.0 software. *p < 0.038 and **p < 0.032.

To confirm the level of *Ld*GS protein expression in mutant cell line, enzyme activity was performed with crude cell lysate of the *Leishmania* cell lines using inorganic phosphate (P_i_) determination method as discussed in methodology section. Higher specific activity of GS in the *Ld*GS^(+/+)^ parasites (311.2 ± 12.4 µmol.min^-1^.mg^-1)^ was seen in comparison to mutants. *Ld*GS^(−/−)^ showed ~82% reduction in specific activity whereas *Ld*GS^(−/−/+)^ showed almost two fold (~46%) reduction in enzyme activity as compared to the *Ld*GS^(+/+)^ (**Figure 3A** **)**.

Growth curve analysis of the five populations at different selected time interval of 24 h was performed for complete five days. During exponential phase of growth at 96 h time point, *Ld*GS^(+/−)^ showed ~1.9 fold reduction in growth. It was observed that in case of *Ld*GS^(+/−/+)^ mutant the parasite numbers was almost same for all selected time point and restored to the levels comparable to that of wild type. *Ld*GS^(−/−)^ failed to grow in media without L-glutamine so was provided with exogenous supplementation of glutamine and showed ~7.0 fold reduction in growth rate compared to wild type cells. *Ld*GS^(−/−/+)^ showed compromised growth pattern but only ~2.2 fold reduction in growth was observed at 96 h time point which indicated recovery of growth **(** **Figure 3B** **).**

It was observed that *Ld*GS^(+/−)^ mutant parasites exhibited almost ~43% reduced *in vitro* infectivity compared to *Ld*GS^(+/+)^. Complete reversal of effect was not observed in *Ld*GS^(+/−/+)^ mutant cell line which showed ~28% reduction in infection. While, *Ld*GS^(−/−)^ mutant showed ~95% reduction of *in vitro* infectivity and replication of intracellular amastigotes compared to wild type [*Ld*GS^(+/+)^] promastigotes. *Ld*GS^(−/−/+)^ mutant parasite showed ~73% reduction in infectivity to macrophages relative to the wild type control. These results indicated that the GS mutant parasites showed impairment in infectivity and survival inside the macrophages and the effect was reversed by complementing with GS expression **(** **Figure 3C** **)**.

### Role of GS Deletion in Parasite Infectivity *In Vivo*

The impact of the deletion of GS in *Leishmania* has been verified for the capacity of its infection, *in vivo*, as only amastigotes are the pathogenic morphs in mammalian hosts. *Ld*GS^(−/−)^ were found most incompetent in infecting the hosts. As evidenced, a reduced parasite-burden was found by 96.4 ± 3.38% (P<0.001) and 77.83 ± 3.52% (P<0.001) in the spleen and liver, respectively, in comparison to the wild type *L. donovani* (MHOM/IN/1980/Dd8) infection. However, the *Ld*GS^(+/−)^ were more infective and the proliferation restriction was found by 47.2 ± 11.69% (P<0.002) and 56.80 ± 8.36% (P<0.003) in the spleen and liver, respectively. Complete reversal of the effect was not achieved in infected animals by *Ld*GS^(+/−/+)^ [inhibition in the spleen by 55.5 ± 2.50% (P<0.001) and liver 67.86 ± 6.89% (P<0.001)] and *Ld*GS^(−/−/+)^ [inhibition in spleen by 39.67 ± 6.26% (P<0.009) and liver 62.03 ± 5.03% (P<0.002)]. The role of GS was further verified for its importance in parasite survival, *in vivo*, by overexpressing it in *L. donovani*. *Ld*GS^(++/++)^ were found efficient over both *Ld*GS^(−/−)^ and *Ld*GS^(+/−)^ as evidenced in comparison to the wild type parasite infection at one-month post infected animals [inhibition only by 23.5 ± 15.05% and 23.97 ± 16.21% in the spleen and liver, respectively] as -represented in (**Figure 3D** I and II). The presence of *Leishmania*-kDNA in respect to Mϕ-GAPDH determined the degree of infection, *in vivo* (Dey et al., 2020). Expression of kDNA further confirmed the result that *Ld*GS^(−/−)^ parasites had low infection rate, *in vivo*, as evidenced from the significantly reduced expression of *Leishmania*-kDNA in *Ld*GS^(−/−)^ infected animals in comparison to *Ld*GS^(+/+)^ at both 15 days and 30 days post infection against normalized equal expression of Mϕ-GAPDH (**Figure 3E**).

### Effect of GS in Parasite Cell Cycle Regulation and Morphology

**Figure 4:**
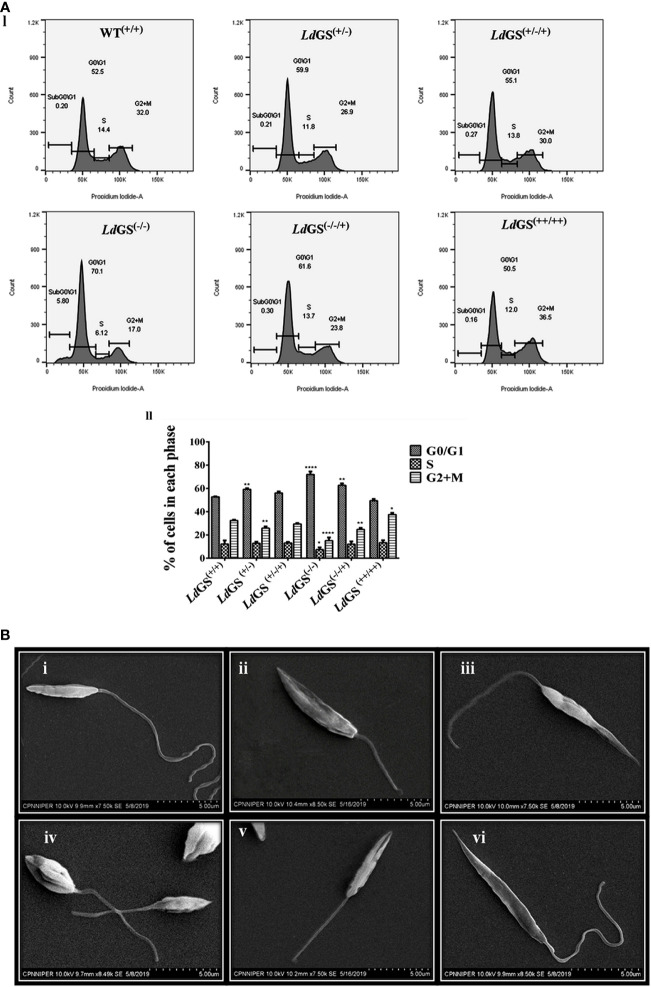
Role of GS in parasite cell cycle and morphology. **(A)** (I) Analysis of DNA content in the wild type and Knockout *L. donovani* parasites after cell synchronization. Fraction of cells in each phase are depicted using histograms. II. Bar graph showing the percentage of cells post synchronization and nutrient supplementation in G_0_/G_1_, S and G_2_/M phases of the cell cycle for wild type and all GS mutant promastigote cell lines. Data represents the mean ± S.D from three independent experiments compared to wild type. *p ≤ 0.05, **p ≤ 0.01, ***p ≤ 0.001 and ****p ≤ 0.0001. **(B)** Morphological alterations of GS mutant promastigotes observed under SEM micrographs (i) SEM micrograph of wild type promastigotes (*Ld*GS^(+/+)^) showing a slender and elongated structure with a healthy flagella, (ii) image of *Ld*GS^(+/−)^ showing cell morphology almost similar to *Ld*GS^(+/+)^ but with shortened flagella, (iii) *Ld*GS^+/−/+)^ complementation mutant cellular morphology almost similar to wild type, (iv) image of null mutant, *Ld*GS^(−/−)^ demonstrating a much altered morphology and a shorter flagella **(v)** add back null mutant, *Ld*GS^(−/−/+)^ show shrinking morphological alteration (vi), *Ld*GS^(++/++)^ mutants show similar healthy appearance like *Ld*GS^(+/+)^ promastigotes and typically more elongated than *Ld*GS^(+/+)^ cells.

Propidium iodide (PI) was used to analyse the DNA content of the cells. The resulting distribution of cells was analyzed by FlowJo software to determine the percentage of cells in G_0_/G_1_, S and G_2_/M phases of the cell cycle. Result suggested that parasites synchronized in the G_1_/S cell cycle phase were able to proceed through cell cycle, except null mutant but percentage of cells in G_2_/M phases was less compared to wild type promastigotes. *Ld*GS^(+/−)^ mutant showed ~1.25 fold less percentage of cells whereas *Ld*GS^(+/−/+)^ mutant showed cell percentage in G_2_/M phases almost similar to *Ld*GS^(+/+)^. *Ld*GS^(−/−)^ mutants were able to complete cell division at a very slow rate and most of the cell population (~70%) was stuck to G_0_/G_1_ phase with entry to S (~ 6.1%) and G_2_/M phases (~17%) of the cell cycle at very low rate. Null mutant showed ~2.1 fold decreased cell population compared to wild type while add back null mutant showed ~1.31 fold decreased cell percentage as demonstrated by flow cytometric analysis (**Figure 4A** I and II).

SEM analysis of *Ld*GS^(+/+)^ and *Ld*GS^(++/++)^ cells showed normal parasite morphology. They had a longer flagellum. The outline was slender and cell surface looked normal (**Figure 4B**).

### Removal of *Ld*GS Gene Increases the Sensitivity of Parasite to Drug, Ultimately Leading to Apoptotic *or* Necrotic Cell Death

**Figure 5:**
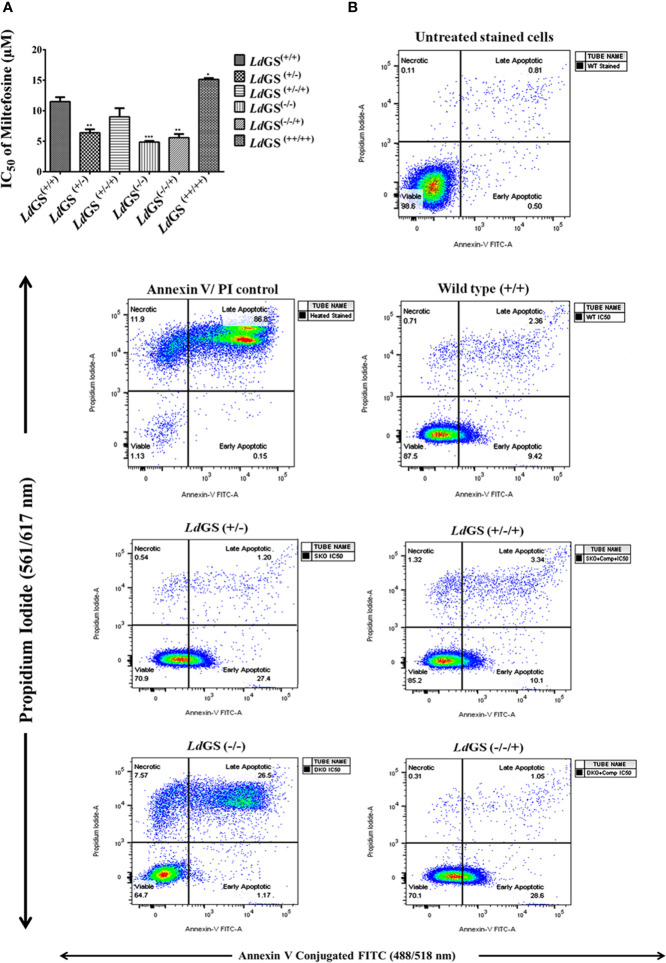
Sensitivity of *L. donovani* glutamine synthetase knockout and overexpressing promastigote parasite to standard drug miltefosine. **(A)** Promastigote growth in the presence of increasing concentration of miltefosine and IC_50_ value of miltefosine was calculated. Data represents the mean ± S.D. from three independent experiments. *p ≤ 0.05, **p ≤ 0.01 and ***p ≤ 0.001. **(B)** Mode of cell death due to action of standard drug in *Leishmania* GS knockout promastigotes. The cells treated with IC_50_ of miltefosine and had been subjected to annexin V-FITC and propidium iodide (PI) staining prior to cell sorting The cells which had not been miltefosine treated but had been dual stained were used as negative control. Flow cytometric data of wild type (*Ld*GS^+/+^), GS heterozygous mutant (*Ld*GS^+/−^), complementation mutant (*Ld*GS^+/−/+^), null mutant (*Ld*GS^−/−^) and add back null mutant (*Ld*GS^−/−/+^) treated with miltefosine followed by 24 h incubation.

Data suggested increase in sensitivity of knockout cells to standard drug. *Ld*GS^(++/++)^ showed ~1.3 fold increase in IC_50_ value of miltefosine (IC_50_:15.1 ± 0.17 µM) compared to *Ld*GS^(+/+)^ cells (IC_50_:11.5 ± 0.70 µM). *Ld*GS^(−/−)^ mutants showed ~2.4 fold decrease in IC_50_ value (IC_50_: 4.8 ± 0.21µM) compared to *Ld*GS^(+/+)^. *Ld*GS^(−/−/+)^ mutant showed reduction in IC_50_ value (IC_50_: 5.6 ± 0.61 µM) comparable to GS heterozygous mutant (**Figure 5A**). The data suggested that GS gene deletion is rendering parasite sensitive to miltefosine and the effect is reversed upon complementation with GS gene. We had tested other antileishmanial drug (Amphotericin B) in our study. DKO and the other knockout strains were not able to sustain the drug selection pressure so further detailed study could not be carried out (data not shown).

Percentage of early apoptotic cells were increased in *Ld*GS^(+/−)^ mutant (27.5%) and *Ld*GS^(−/−/+)^ mutant cells (28.6%) upon treatment with IC_50_ concentration of miltefosine compared to wild type (9.42%) and complementation mutant (*Ld*GS^+/−/+^) cells (10.1%) in FACS analysis performed with Annexin V-FITC and PI. *Ld*GS^(−/−)^ mutants undergo late apoptosis or necrosis upon treatment with similar concentration of miltefosine and the percentage of late apoptotic cells or necrotic cells was found to be 26.5% (**Figure 5B** **).**

## Discussion

Extensive study of the metabolic pathways in *L. donovani* has been done however the knowledge regarding the amino acid metabolizing enzymes in *Leishmania* promastigotes and amastigotes including regulation of the amino acid metabolism throughout the course of infection of the host, is rudimentary. A more detailed knowledge of amino acid metabolic network including uptake, degradation and biosynthesis is quiet essential to enrich our knowledge of Leishmania biology. Exploring the kinetoplastid amino acid metabolism may open a new array of therapeutic targets. One such amino acid metabolic pathway enzyme which has been explored in *Leishmania* parasite in this study is glutamine synthetase. It is a vital metabolic enzyme facilitating many biochemical reactions. It exists in all kingdoms of life including prokaryotes, extra thermophilic archeons, fungi, protozoans, plants as well as in humans.

Previous reports from our lab had identified GS from Leishmania and *in silico* analysis had provided structural aspects of variations in host and parasite enzyme. The biochemical and structural studies had pointed towards its potential as antileishmanial drug target (Kumar et al., 2020). The present study provides molecular and functional evidence of the role of glutamine synthetase in growth and infectivity of *L. donovani* and its essentiality for the parasites. Two strategies were designed to study glutamine synthetase expression by gene replacement and by episomal gene over expression. GS Overexpression was confirmed by immunoblotting and enzyme activity. Overexpression studies revealed increase in growth and infective ability of the parasites. Confirmation of *Ld*GS knockout in transgenic strains at genomic level was done by PCR and changes in *Ld*GS expression at the transcriptional and translational level was studied by RT-PCR, western blot and enzyme activity. Null mutants were generated which could survive only when exogenously supplied with glutamine. In *Ld*GS mutants, the parasite grows slowly and was less infective. Here we show that glutamine limitation hinders the growth of *Ld*GS null mutant, but can be rescued by nutrient supplementation under *in vitro* conditions. Our observations further strengthens the fact that media composition is an important parameter which influences the study of gene knockout of metabolic pathway enzymes (Boitz et al., 2017). There is no evidence till date that *Leishmania* can use host L-glutamine (taken as dietary supplement) for their growth and survivability, however it has been reported that carbon skeletons of the amino acids, especially in promastigotes acts as an energy source. In promastigotes more amino acid utilization is seen than in amastigotes. Glutamine is synthesized using environmental glutamate by interlinked pathway through glycosomal GMP synthase, finally utilized for synthesis of trypanothione (Glew et al., 1988; Subramanian and Sarkar, 2017).

The effect of GS gene deletion on *in vitro* infectivity of monocyte differentiated macrophage was observed. Among GS knockouts, heterozygous mutant parasite [(*Ld*GS (^+/−^)] had almost 56% reduction in infectivity while null mutant [(*Ld*GS (^−/−^)] showed ~93% of drastic reduction in infectivity to macrophage as compared to wild type cells. On episomal expression of GS gene in heterozygous mutants [(*Ld*GS (^+/−^/+)] parasite infectivity was restored almost comparable to wild type. Unlike null mutant, GS add back null mutant [(*Ld*GS (^−/−^/+)] parasites showed less alteration in the growth pattern and infectivity as compared to null mutants. Therefore, it may be said that the knockout parasite attempts to recover when episomal complementation is done. However, complete recovery is not seen. Residual *Ld*GS activity of null mutant is probably due to the fact that, apart from GS there are several other ATP dependent enzymes in total cell lysate that catalyzes dephosphorylation of ATP which results in formation of blue color phosphomolybdate complex. GS first needs ATP to get active catalytic confirmation which facilitates binding of Glutamate that finally result in release of inorganic phosphate and Glutamine. Glutamine has been known to modulate immune response and play essential role for the control of *L. donovani* infection to macrophages. The gene of glutamine metabolism have been found to be upregulated in macrophages infected by *L. donovani*, as revealed by transcriptomic analysis (Ferreira et al., 2020). It is known that the protein expression is more when the gene integration happens in the genome, as compared to episomal addition. This data correlates with previously reported result in case of *T. cruzi* where GS was reported to be responsible for host-cell invasion establishment of infection (Crispim et al., 2018). In *T. cruzi*, GS facilitates cellular replication and infection to host cell by helping it escape parasitophorous vacuole. This established the link between GS activity and how the parasite grows and establishes infection inside host (Crispim et al., 2018). GS is involved in pathogenesis of *M. tuberculosis, S. typhimurium, S. suis, S. pneumonia*e and *S. enterica* (Klose and Mekalanos, 1997; Harth et al., 2000; Hendriksen et al., 2008; Si et al., 2009; Aurass et al., 2018). Also *gln*A-1 mutant strain of *M. tuberculosis* has less ability to infect THP-1 cells (Tullius et al., 2003). Glutamine has been found to regulate virulence gene expression responsible for invasion of host cell by *T. gondii* and *L. monocytogenes* (Lee et al., 2014; Haber et al., 2017). Similar result was obtained in *in vivo* infection with knockout strains in mice. Parasite burden was checked in spleen and liver of infected mice. However, low parasitic burden in *Ld*GS (^+/−^/+) mutant than *Ld*GS (^+/−^) mutant was observed. The parasites that overexpress *Ld*GS are more infective and grow better *in vitro*, but *in vivo*, mice infected with parasites that overexpress the enzyme do not present increased parasite load compared to animals infected with WT parasites. The outcomes of infectivity, *in vitro* and *in vivo* may vary due to the presence of different cytokine milieu that can affect infection vividly. Active segregation of episomal vectors does not take place and so, the daughter cells do not inherit equal copy number of the constructs. All transfected parasites do not have the same number of copies due to which their expression varies from cell to cell. Therefore, if the copy number is too high, it may be toxic to parasite. If the copy number is too low, it may not be enough to complement gene deletion. Episomal vectors lack active segregation mechanisms so possibly distribution between daughter cells is not always equal. Furthermore, the gene expression level from an episome cannot be controlled and fluctuates among transfected parasites depending upon the copy number of episome. Thus, the target protein expression may be low and insufficient for rescue or too high and have deleterious effects on parasite growth (Kushnir et al., 2011; Roberts, 2011). Cell cycle experiment with synchronized cells shows that glutamine is essential and is required for progression through the restriction point in mid-to late G1 and through S phase into cell division (G_2_/M). For cell cycle progress to mid to late G_1_, glucose and glutamine both are essential. But only glutamine is required for transition through S phase (Colombo et al., 2011). Amino acid metabolism plays an important role in critical life processes of parasites such as pathogens and resistance to stress inside the host. In critical biological processes during the life cycle of these parasites, such as the establishment of infection within the mammalian and insect hosts, differentiation, modulation of the cell cycle and resistance to extreme stress conditions metabolism of amino acid play very important role (Marchese et al., 2018). Morphological changes were observed in GS mutant cells which can be inferred to the fact that either *Ld*GS maintains the cell morphology or mutating the gene pushes the cells under biochemical and oxidative stress which in turn affects the morphology.

**Figure 6:**
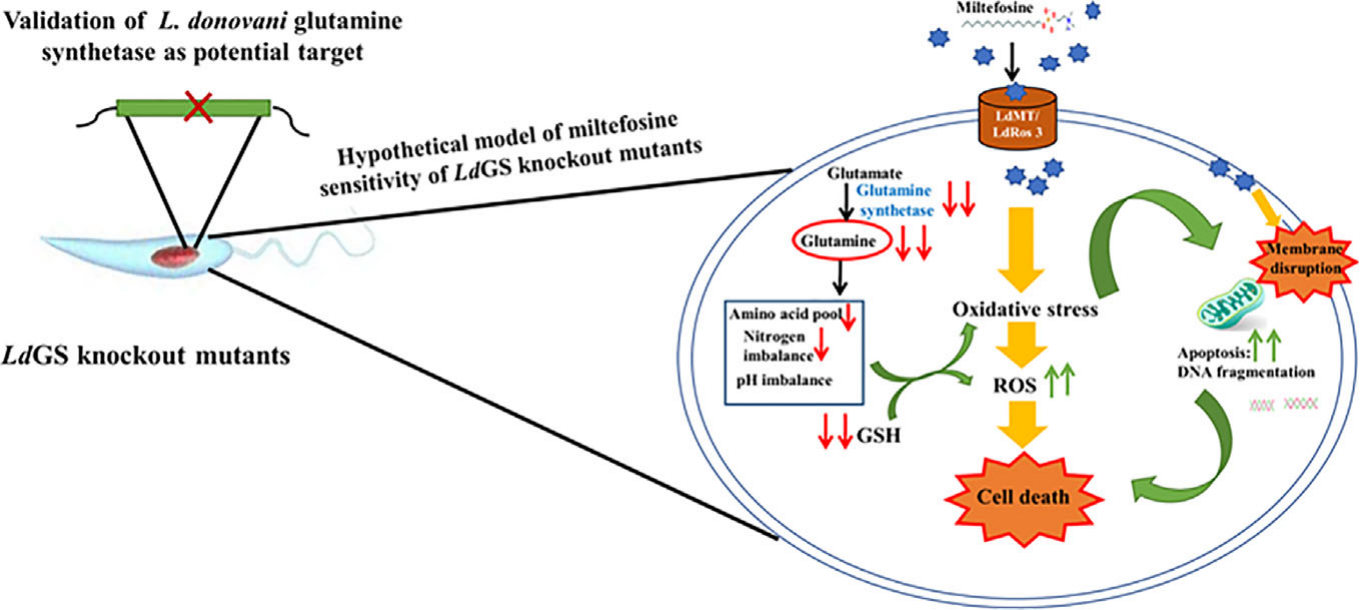
Hypothetical model explaining sensitivity of *Ld*GS knockout strains to Miltefosine. Based on experimental results a model was proposed to understand the link between GS expression and miltefosine sensitivity.

GS knockout made *L. donovani* parasites more sensitive to standard drug miltefosine and mode of cell death was evaluated by annexin V-FITC/PI staining. Wild type parasites were unstained whereas IC_50_ treated cells showed less population of early apoptotic cells stained with annexin V-FITC (externalization of phosphatidylserine). Assessment of flow cytometric data of null mutants treated with similar drug concentration showed significant population of cells undergoing late apoptosis or necrosis which were stained by both annexin V and PI. GS null mutant pre-treated with NAC (N -acetyl cysteine) showed recovery of cells with a smaller number of both annexin V and PI-stained cells indicating role of oxidative stress leading to apoptotic signals (externalization of phosphatidylserine) in GS null mutants compared to wild-type cells. Glutamine synthetase is also essential for combating oxidative stress as the GS null mutants upon miltefosine treatment were observed to have higher sensitivity to miltefosine as compared to wild type cells. Treatment time with miltefosine vary in case of both cell based study and FACS data. It was 48 h in case of cell-based study while in case of FACS based analysis cells were incubated with drug for 24 h. Incubating cells with drug for 48 h resulted in cellular morphological alteration and large number of dead cells specially in case of null mutants which made the comparative analysis of data with different cell lines difficult. The hypothetical model of miltefosine sensitivity of *Ld*GS mutants is depicted in **Figure 6**.

Overall findings of our data provide the role of GS gene overexpression and deletion of alleles leading to alteration in the growth pattern and infectivity of promastigotes. This work deals with the impact of GS deletion of *L. donovani* on the survivability and infectivity of the parasites, *in vitro* and *in vivo* conditions. The GS null mutant parasites failed to grow in glutamine deprived media and could survive only when maintained in glutamine supplemented media, pointing towards the essential role of this gene in *Leishmania* parasites. Hence, experimental results indicate that *Ld*GS is pivotal to cell survival and its metabolic regulation. The above studies validate the conserved glutamine synthetase as promising therapeutic target and can be used for *Leishmania* specific designing of new drug candidates.

## Data Availability Statement

The original contributions presented in the study are included in the article/**Supplementary Material**. Further inquiries can be directed to the corresponding author.

## Ethics Statement

The study was performed in accordance with appropriate institutional guidelines for animals. All animal experiments were approved by the Institutional Animal Ethics Committee, WBSU, Barasat [File no. WBSU/IAEC/2019-20/8(CP), dated 20.05.2019]. IAEC registration no. 1394/GO/Re/S/10/CPCSEA, dated 09/12/2016) approved by, Committee for the Purpose of Control and Supervision of Experiments on Animals, Ministry of Environment and Forest, Govt. of India.

## Author Contributions

SS and CP conceived and designed the experiments. VK, SG, and KR performed the research. SS and CP wrote the manuscript. All authors contributed to the article and approved the submitted version.

## Funding

We acknowledge the DST-FIST, Govt. of India [Ref: SR/FST/LS1-001/2014] and DBT-BOOST, Govt. of West Bengal [Ref: 49 (11)/BT (Estt)/1P-4/2013 (Part-1)] for providing the research infrastructures in WBSU, Barasat. VK received SRF in the project funded by Department of Pharmaceuticals, India [KA-01(2013-14)-Sushma]. SG received SRF from ICMR, Govt. of India [Ref: Fellowship/65/20I9-ECD-II dated 17/07/2019], and KR received the fellowship from INSPIRE-Department of Science and Technology, Govt. of India [Ref: Dy. No. C/3321/IFD/2019-20 dated 25/09/2019].

## Conflict of Interest

The authors declare that the research was conducted in the absence of any commercial or financial relationships that could be construed as a potential conflict of interest.

## Supplementary Material

Supplementary Figure 1 The 5′UTR and 3′UTR sequences of *Ld*GS used for cloning in knockout vectors are depicted.

Supplementary Figure 2
**(A)** Vector map of pXG Hygro, used to generate heterozygous construct **(B)** Vector map of pXG-Neo, used to generate null mutant construct **(C)** Vector map of pXG-Phleo, used to generate complementation construct (kind gift from Prof. Stephen M. Beverley, Department of Molecular Microbiology, Washington University, S. Louis, USA). **(D)** Vector map of psp72αhygroα, used to generate overexpression construct (kind gift from Prof. Rentala Madhubala, JNU, New Delhi).

Supplementary Figure 3 Scheme for GS gene Knockout in *L. donovani* parasites. **(A)** The Knockout was done using targeted gene replacement(UTR represents untranslated region); **(B)** Schematic representation to highlight set of primers along with their position of binding for confirmation of appropriate integration of *Ld*GS gene replacement cassette with the genes for neomycin resistance (*neo^r^* **-**neomycin phosphotransferase) or hygromycin resistance (*hygro^r^*-hygromycin phosphotransferase) and 5′ and 3′ UTR were present in flanking region.

Supplementary Figure 4 Comparison of the growth rate of all knockout strains in glutamine deprived media.

Supplementary Table 1 Primers used for generating *Ld*GS overexpressor and knockout constructs.

Supplementary Table 2 List of primers used to confirm appropriate integration of *Ld*GS knockout cassettes.

Supplementary Table 3 List of primers used for RT-PCR analysis of GS mutant parasite.

Supplementary Table 4
**(A, B)** Parasite load in infected male BALB/c mice **(A)** Spleen **(B)** Liver of mice infected with different mutant strains of *L. donovani* Dd8, *in vivo*. Data are representative of Mean ± SE from three independent experiments with 5 animals per group.
