# Supplementary material for: Deletion of Glutamine Synthetase Gene Disrupts the Survivability and Infectivity of Leishmania donovani
Source: Front Cell Infect Microbiol. 2021 Feb 26;11:622266. doi: 10.3389/fcimb.2021.622266 (PMC7959746; doi:10.3389/fcimb.2021.622266)
Supplement: Supplementary Figure 1 — The 5′UTR and 3′UTR sequences of LdGS used for cloning in knockout vectors are depicted. [file DataSheet_1.doc]

**Supplementary Figures and Tables**

Deletion of glutamine synthetase gene disrupts the survivability and infectivity of Leishmania donovani

Vinay Kumar^1^, Sanhita Ghosh^2^, Kamalika Roy^2^, Chiranjib Pal^2^, Sushma Singh^1*^

^1^Department of Biotechnology, National Institute of Pharmaceutical Education and Research, SAS Nagar, Mohali-160062, Punjab, India.

^2^Cellular Immunology and Experimental Therapeutics Laboratory, Department of Zoology, West Bengal State University, Barasat, 24 Parganas (North), Pin- 700126, West Bengal, India

* Corresponding author at Department of Biotechnology, National Institute of Pharmaceutical Education and Research, SAS Nagar, Mohali-160062, Punjab, India. Tel.: +91-172-2292208; fax: +91-172-2214692.

E-mail address: [***sushmasingh@niper.ac.in***](mailto:sushmasingh@niper.ac.in) (Sushma Singh).


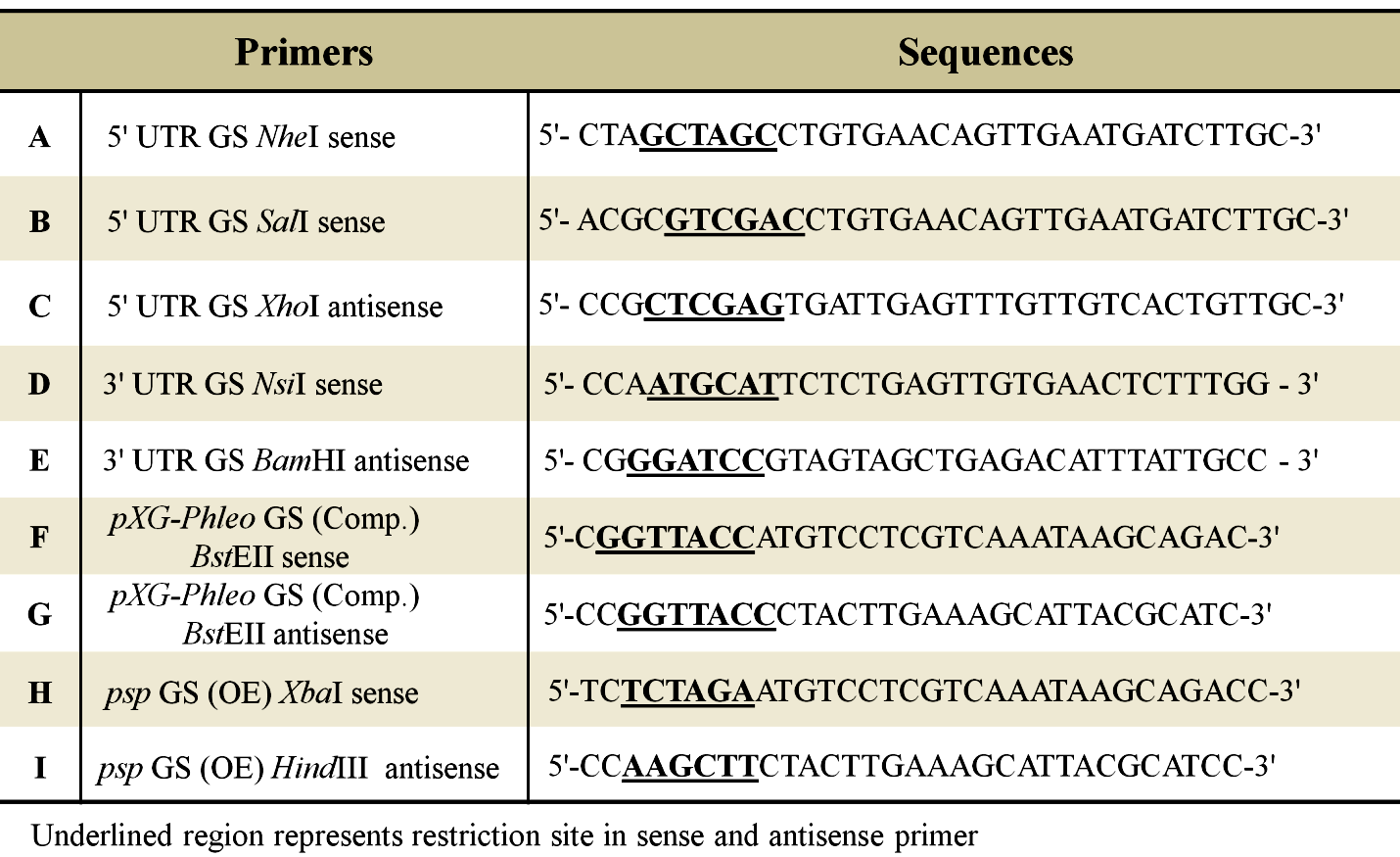


**Table S1.**


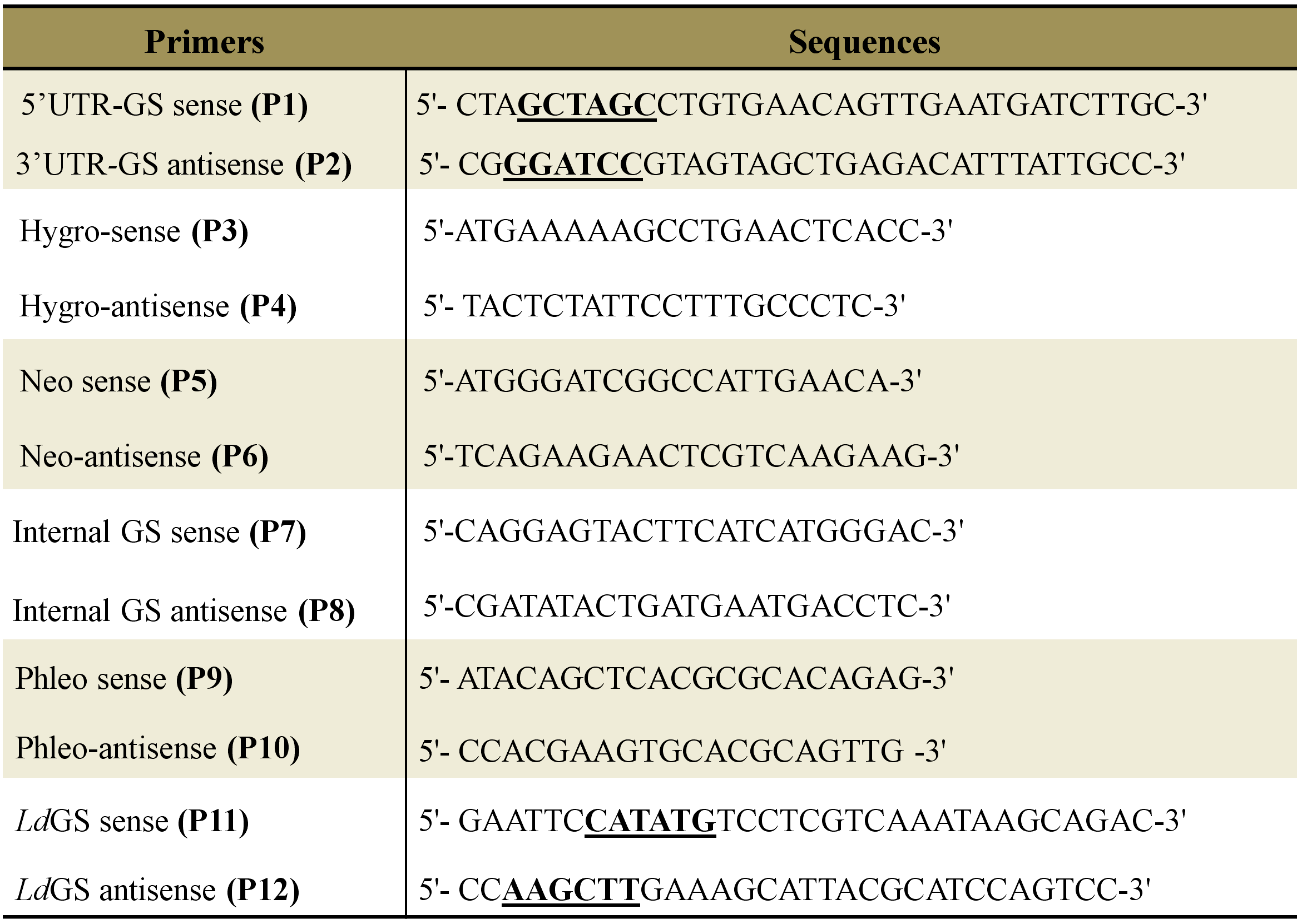


**Table S2.**


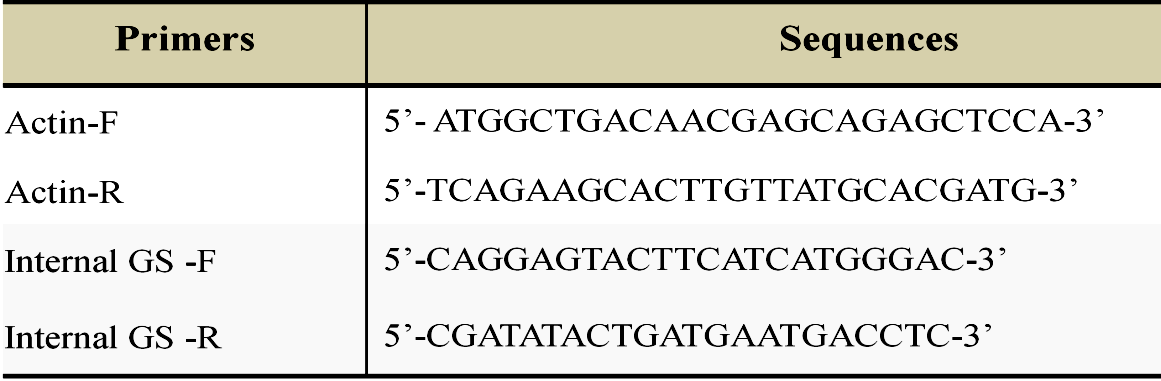


**Table S3.**

| **BALB/c infected with** | **Parasite burden, Spleen (×10^7^)**  **Mean ± SE** | ***P* values [vs *Ld*GS^(+/+)^]** |
| --- | --- | --- |
| ***LdGS^(+/+)^*** | 79.66 ± 8.41 | NA |
| ***Ld*GS^(+/-)^** | 40.33 ± 6.64 | <0.002 |
| ***Ld*GS^(+/-/+)^** | 35.33 ± 3.84 | <0.001 |
| ***Ld*GS^(-/-)^** | 6.0 ± 2.08 | <0.001 |
| ***Ld*GS^(-/-/+)^** | 47.0 ± 0.57 | <0.009 |
| ***Ld*GS^(++/++)^** | 62.66 ± 5.78 | 0.318 (not significant) |
| **Vc** | 39.33 ± 5.20 | <0.002 |

**Table S4 A**

| **BALB/c infected with** | **Parasite burden, Liver (×10^7^)**  **Mean ± SE** | ***P* values [vs *Ld*GS^(+/+)^]** |
| --- | --- | --- |
| ***LdGS^(+/+)^*** | 330 ± 51.96 | NA |
| ***Ld*GS^(+/-)^** | 134 ± 4.0 | <0.003 |
| ***Ld*GS^(+/-/+)^** | 100.0 ± 10.0 | <0.001 |
| ***Ld*GS^(-/-)^** | 70.0 ± 5.77 | <0.001 |
| ***Ld*GS^(-/-/+)^** | 120.0 ± 5.8 | <0.002 |
| ***Ld*GS^(++/++)^** | 246 ± 46.66 | 0.386 (not significant) |
| **Vc** | 126.66 ± 16.66 | <0.002 |

**Table S4 B**

5’UTR GS (Sequence- 737 bp)

CTGTGAACAGTTGAATGATCTTGCGGTTCCTTTGGCGACGGCTGACATGCGCAGGGGGGCTGTGGCGGGCGCAAAGGAGGGGAGAGGGGAACATAGGGATTATTGCCGTGAACTCTTTTTTTTCCAGACCACAACGCTGCGGATGAGCTGCTGAAGCGGGTCGATGATATCGAGTGGACAGTTTATCACAACGACACATTTCTATCCAAATAGCTTGCTTTTCAGGTGGGTAAATGTGGCTTCGCGTGTTGACAGATATTGAATAGCATGCCGAGAAAGCTGATGAAAGCAGCTGTGGATGCGGTGGAAGTGATGGACTGTTCTGGAATCGCTTTTCGTTCGACTGGAAAATCAATTTCCGTAAACATCTTCAGAAGCTGCCGCAAAGCCGATCCCACTGAACTTTTTTCCCATTTGCCAAGTAATGAAGTCTATGGGTGACAGACACACACAGAGGAGTGAGAGAGGATTTGAAAGGCGGCATTGGGTTTGTGTGCGTATGCTGCGAGGCGGACCTGCTACACATGAATCTCGGTGCAGCTTTCTTGGCTGCACTTGGTTGCTTGACATACTCCCTCATTTCTGCCACTCTTCCTTCAATCACGTCCTTTTTTTCAACCGCTATAAGTATCAACAGGTAGTGTGGTATCCACTCCTAAGGCTGGGTGATTGGCTAAAAGCGCATTGCTCTTCTCCTACTACCGACAACGCGGCAACAGTGACAACAAACTCAATCA

3’UTR GS (Sequence- 785 bp)

TCTCTGAGTTGTGAACTCTTTGGTCGACTTATCCGTGTGACGAAAGTTCTATTTTAAATTGCTTTGGCGTATTTGAATTTGATACCTTGTCCGTTTCCAAATCGTATTTACAACATCAATACCGATCTTCGGCAGAGTTAGGGGGCACAAAGAAACACCGGCGAACAAAAAGTATGTAAAGGATGACAGATAAATGATTCGCTTCTTGTACGGCTACTATTGTGACTCTTTATTGAGACTCAAGTGTGCTCAGCCAGTGCGAGAATTTTTGCCGTTATAATTAAAAAAAAACGGTGTGTTCGCATCTCTGTCATATAAAAGTCTTTTTTTTTGCGTTGTTTTTTTGTTTCCTTTTTATCATTCACCCTTTAACCAAAAGAACCTATTTTTCGCTATGACCCAACTTGATTGAGCCGTTGGGCATAGAGACGGGCTTTGTCTTGATTGTTTAAAAGGAGTGAGGTCTGTGAAAAGTAACACAACCGTGTATGCTGTAGTAAGTTTGCTTTTTTTTTTCGTTCCTCAAGGAGGAGTCCCATCGATAGCGTCTCTTTTCGGTACCGTTGAGAATGTTTGGGGTCGAATTACCAATTGAGAGAAACTGACAGGCAGTGGCCCTGAATGCTGAAGTATTGAACGCTAACGGCGTCCTTTGCTCAGGCATACTTTTTTTTTTTTACGATGCGCCATACCTTGATGAGGACGAAGGCGGTGGCTGCTACTCACTCCAATGAGTTTGGGGTTTGTACTGCAATGTTGTTGGCAATAAATGTCTCAGCTACTAC

**Supplementary Figure S1**


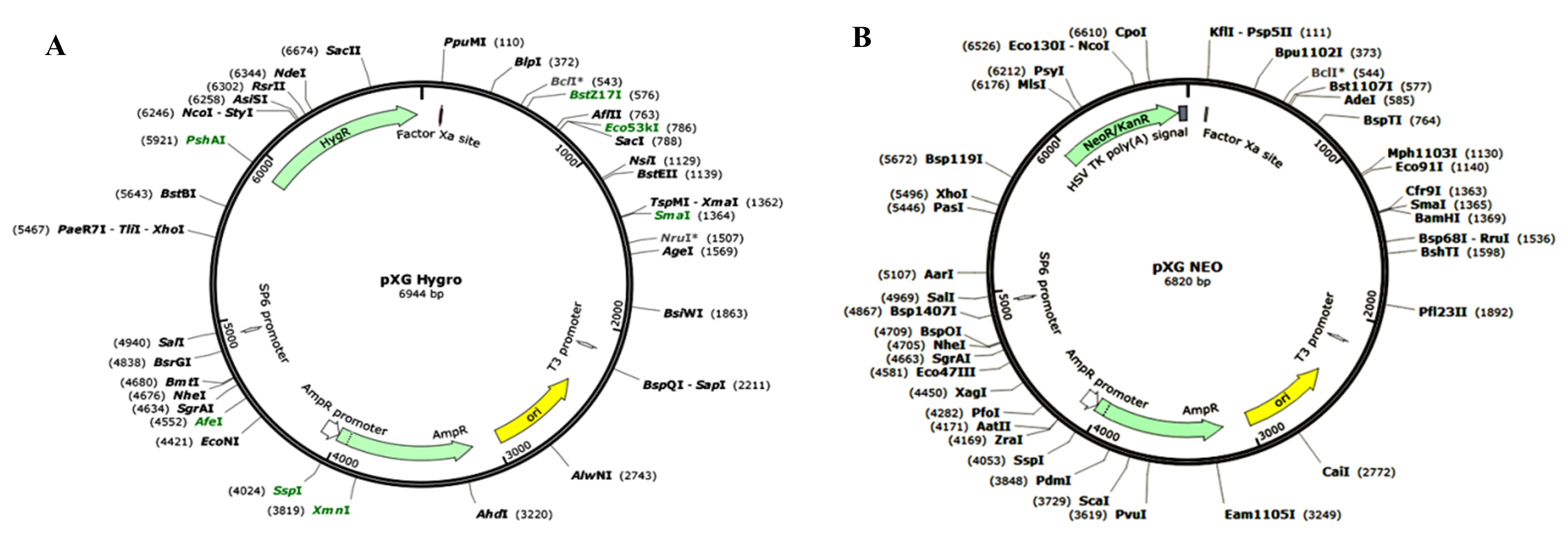


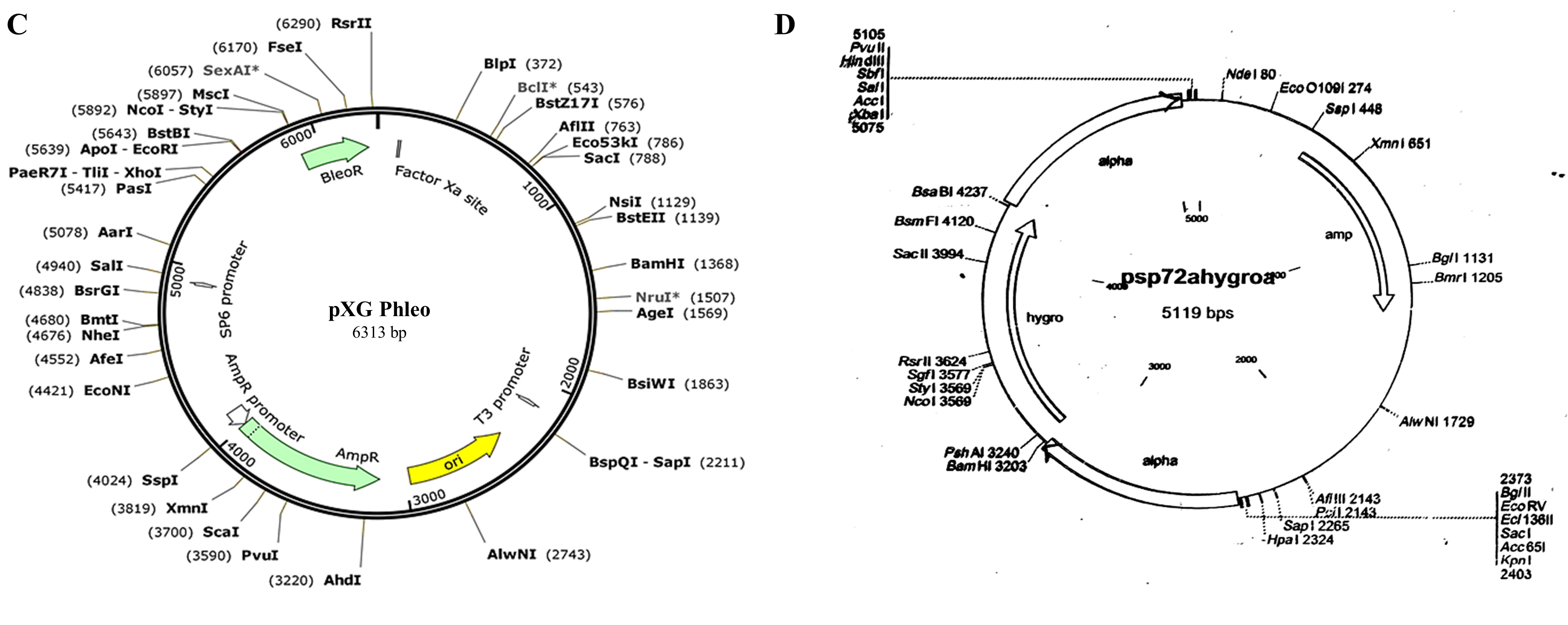


**Supplementary Figure S 2**


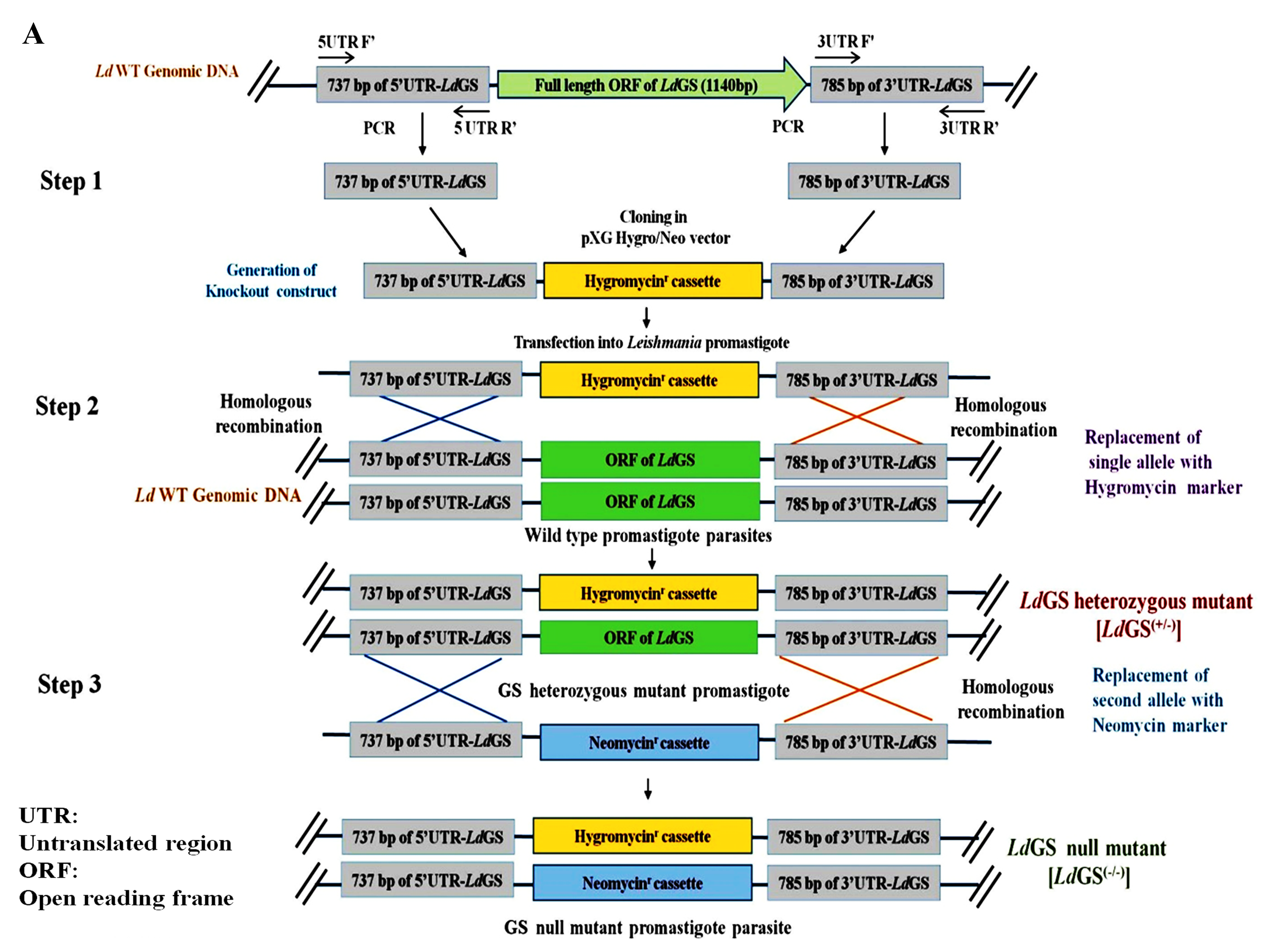


**Supplementary Figure S 3A**


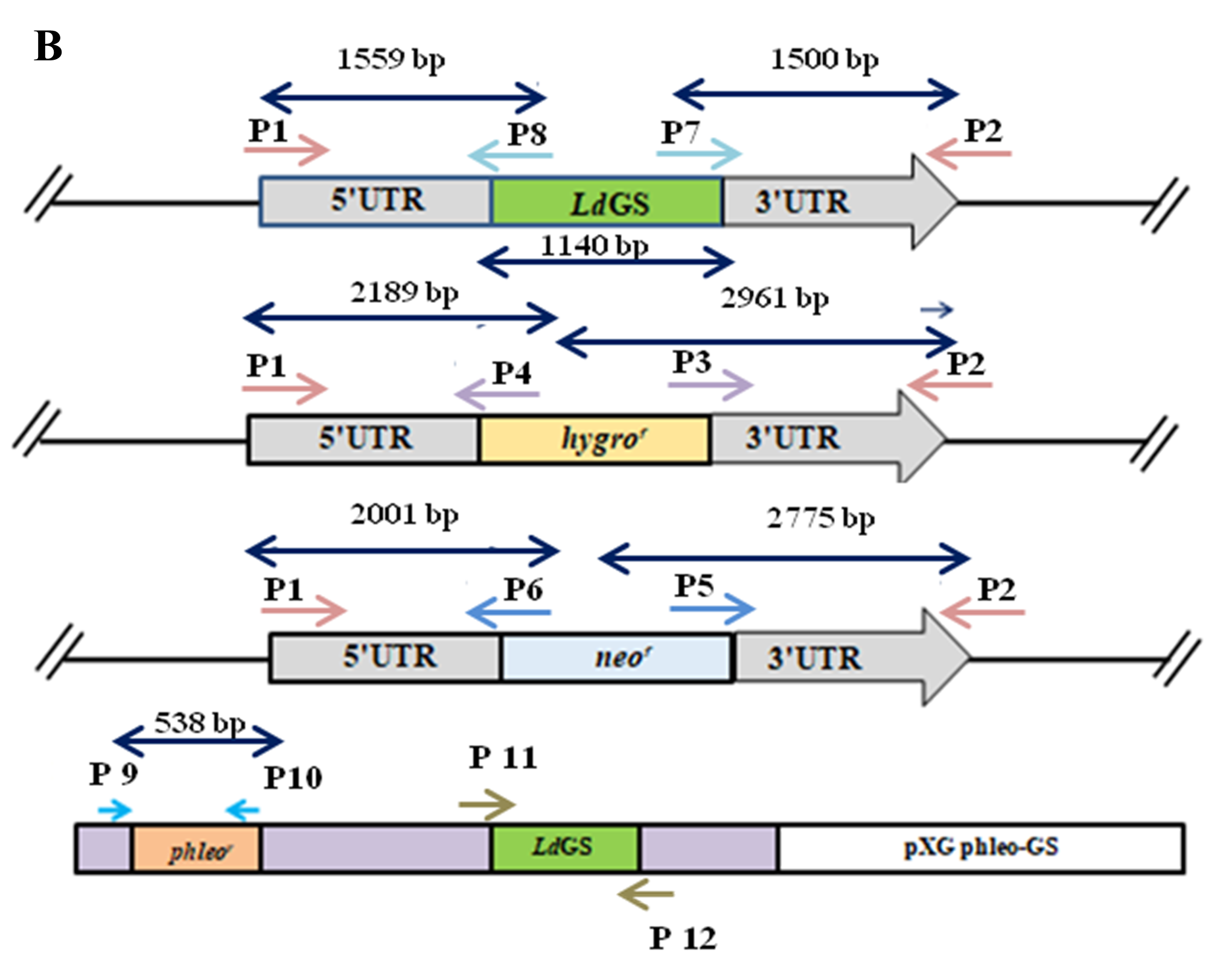


**Supplementary Figure S 3B**


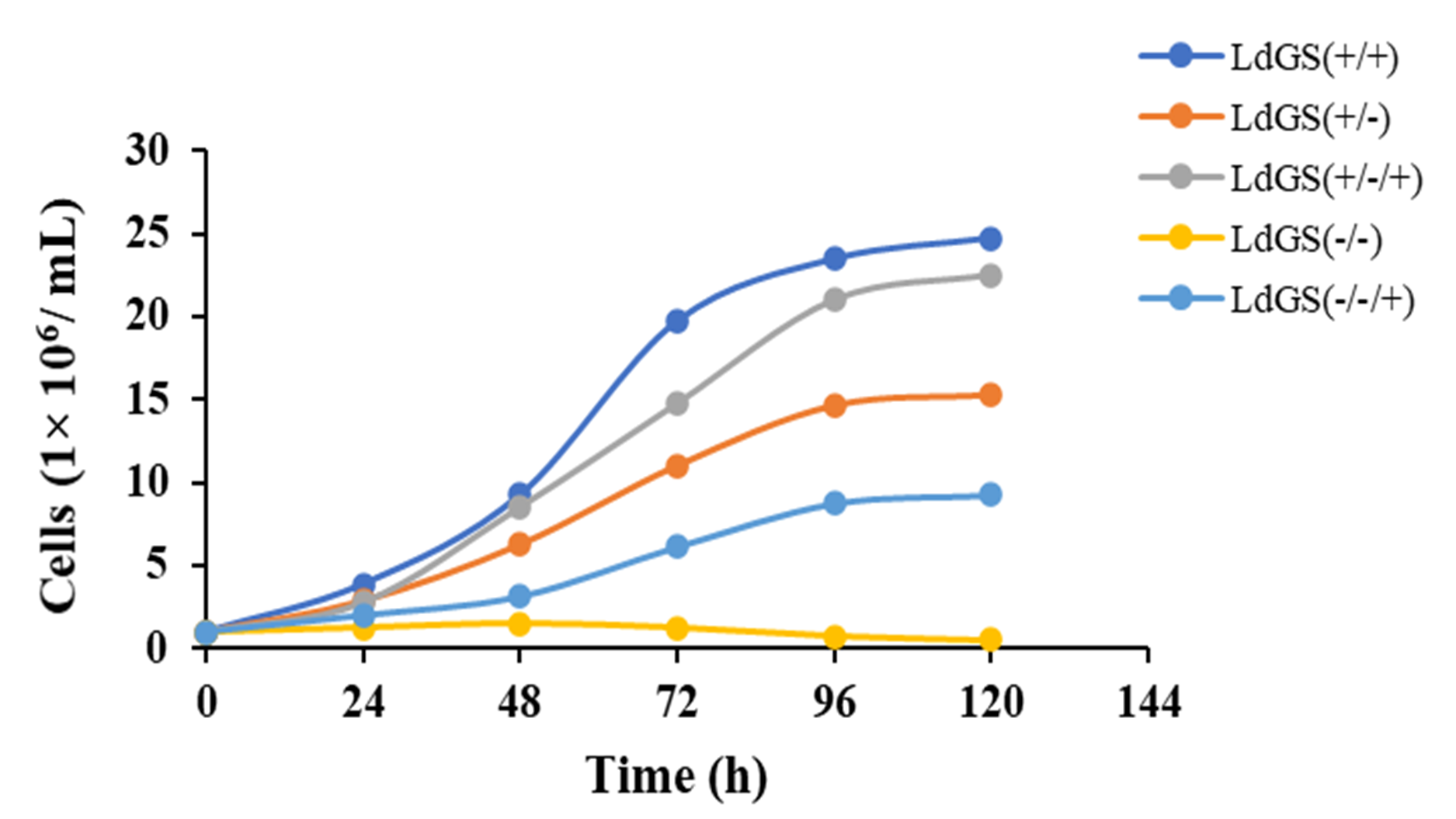


**Supplementary Figure S 4**
